# Stable overexpression of native and artificial miRNAs for the production of differentially fucosylated antibodies in CHO cells

**DOI:** 10.1002/elsc.202300234

**Published:** 2024-04-01

**Authors:** Patrick Schlossbauer, Lukas Naumann, Florian Klingler, Madina Burkhart, René Handrick, Kathrin Korff, Christian Neusüß, Kerstin Otte, Friedemann Hesse

**Affiliations:** ^1^ Institute for Applied Biotechnology University of Applied Sciences Biberach Biberach Germany; ^2^ Department of Chemistry Aalen University Aalen Germany

**Keywords:** biopharmaceuticals, biosimilar, CHO, fucosylation engineering, microRNA

## Abstract

Cell engineering strategies typically rely on energy‐consuming overexpression of genes or radical gene‐knock out. Both strategies are not particularly convenient for the generation of slightly modulated phenotypes, as needed in biosimilar development of for example differentially fucosylated monoclonal antibodies (mAbs). Recently, transiently transfected small noncoding microRNAs (miRNAs), known to be regulators of entire gene networks, have emerged as potent fucosylation modulators in Chinese hamster ovary (CHO) production cells. Here, we demonstrate the applicability of stable miRNA overexpression in CHO production cells to adjust the fucosylation pattern of mAbs as a model phenotype. For this purpose, we applied a miRNA chaining strategy to achieve adjustability of fucosylation in stable cell pools. In addition, we were able to implement recently developed artificial miRNAs (amiRNAs) based on native miRNA sequences into a stable CHO expression system to even further fine‐tune fucosylation regulation. Our results demonstrate the potential of miRNAs as a versatile tool to control mAb fucosylation in CHO production cells without adverse side effects on important process parameters.

**Abbreviations**
ADCC: antibody‐dependent cellular cytotoxicity
amiRNA/amiR: artificial miRNA
CHO: Chinese hamster ovary
D‐Gal: D‐Galactose
D‐Glc: D‐Glucose
D‐Man: D‐Mannose
Fru‐1‐P: fructose‐1‐phosphate
FUK: fucokinase
FUT8: fucosyltransferase 8
Gal‐1‐P: galactose‐1‐phosphate
GAPDH: glyceraldehyde‐3‐phosphate dehydrogenase
GDP‐Fuc: GDP‐Fucose
GFP: green fluorescence protein
Glc‐1‐P: glucose‐1‐phosphate
HPLC: high‐performance liquid chromatography
IgG: immunoglobulin G
LC‐MS: liquid chromatography coupled with mass spectrometry
mAb/mAbs: monoclonal antibody/antibodies
Man‐1‐P: mannose‐6‐phosphate
Man‐6‐P: mannose‐1‐phosphate
miRNA: microRNA
mRNA: messenger RNA
NT: non‐targeting control siRNA
PC: principal component
PCA: principal component analysis
pre‐miRNA: precursor miRNA
pri‐miRNA: primary miRNA
QC: quality control standard
qPCR: quantitative reverse transcription polymerase chain reaction
RNAi: RNA interference
SD: standard deviation
siRNA: small interfering RNA
UT: untransfected cells
UTR: untranslated region
vc: viable cells
VCC: viable cell concentration
WT: wild type miRNA

## 1 INTRODUCTION

Chinese hamster ovary (CHO) cells are widely used in biotechnology as hosts for the production of recombinant proteins, including monoclonal antibodies, and enzymes [1, 2]. Often, CHO cells are genetically engineered to improve their growth or production characteristics. Usually, this is achieved using classical methods, such as knock‐out or overexpression of genes [3, 4, 5]. However, while the deletion or overexpression of genes only allows to switch gene expression on or off, a more precise fine‐tuning of phenotypic changes is often desired, for example, for the control of protein glycosylation in biosimilars. In these cases, the possibility to modulate the degree of gene expression using RNA interference (RNAi) via small‐interfering RNAs (siRNAs) or micro‐RNAs (miRNAs/miRs) offers an attractive alternative for cell engineering [3, 4, 6, 7, 8]. Both molecules are 20–25 base pairs long, double‐stranded, and lead to mRNA cleavage or repression of protein translation by different modes of action [9, 10, 11, 12, 13, 14]. In contrast to highly target‐specific siRNAs, miRNAs can target hundreds of genes. Therefore, the use of miRNAs as engineering tools can be challenging, but offers the opportunity to control entire cellular pathways without any energy‐consuming translational burden [15]. Therefore, miRNA‐associated engineering approaches were established as efficient tools in CHO cell line engineering for the improvement of productivity, fine‐tuning of product quality, the enhancement of cell growth and survival, and the modulation of metabolic pathways [3, 6, 8, 16, 17, 18, 19, 20, 21, 22, 23, 24, 25, 26, 27, 28, 29, 30]. However, quality‐relevant characteristics of therapeutic proteins like antibody fucosylation have so far been engineered using standard techniques like knock‐out of genes [5, 31, 32, 33, 34], siRNA mediated transient gene knock‐down [27, 35, 36] or miRNA‐mediated transient gene knock‐down [37] only. Regulation of relevant enzymes by the addition of inhibitors [38, 39, 40] or coexpression of intrabodies are also described [41].

In mammalian cells, miRNA biogenesis involves transcription of primary miRNA (pri‐miRNA) by its own promoter (canonical pathway) or from a mirtron (noncanonical pathway), processing of the pri‐miRNA into a precursor miRNA (premiRNA), and trimming into the regulatory active mature miRNA [42, 43, 44, 45, 46, 47]. However, basic research studies of miRNAs typically rely on transient transfection of synthetic ready‐to‐use double‐stranded miRNA mimics, representing mature miRNAs [48, 49]. While this approach offers the great advantage to screen large numbers of molecules in a short time for their initial cellular effects, it lacks applicability in industrial bioprocesses [18, 37]. Furthermore, the mode of action or target affinity appears to be dose‐sensitive and the high concentrations of mature miRNAs in screening experiments might not be achieved in a stable expression context [50, 51]. Nevertheless, in order to enable the applicability of this technology in production processes for biopharmaceuticals, expression plasmids must be used to stably integrate the miRNA sequence into the genome, followed by the exploitation of the native biogenesis pathway. Efficient stable overexpression is highly dependent on several factors such as the plasmid system [23, 52], the genomic integration site [53, 54, 55], and the combination of cell line, medium, and transfection reagent [56, 57, 58]. Plasmid system engineering for a stable miRNA expression include the optimization of miRNA flanking regions or promoters [23, 29, 52], hairpin structures [59], spacer sequences [60], and chaining of multiple miRNAs [6, 21, 23, 59, 60, 61, 62, 63, 64, 65]. Accordingly, stable overexpression of miRNAs bears the risk of an insufficient regulation to trigger a desired biotechnologically relevant phenotypic change [23, 50].

Therefore, we investigated several approaches to achieve and improve phenotypic effects which can be accomplished by the stable expression of miRNAs in a monoclonal antibody (mAb) producing CHO cell line. To this end we engineered the fucosylation of a secreted mAb, as it reflects one of the most important critical quality attributes for therapeutic mAbs and can therefore serve as a suitable model for the generation of biosimilars [5, 37, 66]. Using this model, we aimed to transfer our knowledge of transient miRNA‐based regulation [37] to stable cell line engineering, and tested optimization strategies to improve mAb fucosylation in order to give a potential outlook for the applicability of the technology in biosimilar development. In this context, we also implemented stably expressed artificial miRNAs (amiRNAs/amiRs) as improved engineering tools and performed a small‐scale metabolite analysis to better understand the effects of miRNA‐mediated regulation on the metabolic level of fucosylation precursors in CHO cells.

## 2 MATERIAL & METHODS

### 2.1 Cell culture

Maintenance cultures of suspension‐adapted monoclonal antibody (IgG) producing CHO‐K1‐mAb1 cells (Rentschler Biopharma property) with a reported productivity of >1 g/L [67], were grown in Tube Spin Bioreactor50 tubes (TPP Techno Plastic Products AG, Switzerland) in ProCHO 5 culture medium (Lonza, Belgium), supplemented with 4 mM L‐glutamine (Lonza, Belgium). If not otherwise mentioned, standard cell culture conditions for all experiments were 37°C, 5% CO_2_, and 85% rH at 140 rpm (25 mm orbit) (Kuhner Shaker GmbH, Germany). Viability parameters were routinely checked using a CEDEX XS cell counter (Roche Diagnostics, Germany) with a trypan blue (Thermo Fisher, Germany) exclusion assay. Cells were passaged every 3 days to a starting cell concentration of 2 × 10^5^ vc/mL.

### 2.2 Artificial miRNAs

For stable cloning, amiRNAs were selected based on previously described transient experiments [37]. Briefly, for the design, the wild type (wt) sequences of miR‐34a‐5p and miR‐669h‐5p were adapted in silico towards stronger binding to their respective mRNA target fucosyltransferase 8 (FUT8) by changing multiple bases in the miRNA sequence. AmiRNA mimics and amiRNA encoding plasmids were transfected as described in Section 2.4 and 2.5. Mature amiRNA sequences are given in the supporting information (Table S1).

### 2.3 Plasmids

For miRNA and amiRNA overexpression, the commercial BLOCK‐iT Pol II miR RNAi Expression plasmid Kit with the pcDNA6.2‐GW/EmGFP‐miR plasmid was used (Thermo Fisher Scientific, Germany), following the standard kit protocol. Briefly, the mature miRNA sequence was synthesized in a guide strand together with a fully complementary passenger strand with two deleted nucleotides to form a bulge (Eurofins Genomics Gene Synthesis, Germany). Both strands were annealed to form a duplex with overhangs and ligated into the linearized plasmid harboring flanking regions derived from miR‐155 for proper miRNA processing. The pcDNA6.2‐GW/EmGFP‐miR‐neg. control provided in the kit was used as a mock plasmid. A published FUT8 siRNA [27] was cloned into the plasmid to obtain a positive control for the metabolite analysis experiment. Chaining of miRNAs was performed according to the strategy suggested by the vendor's protocol. In brief, two or four copies of the miRNA were cloned sequentially using a combination of *BglII/XhoI* restriction sites to obtain the backbone and *BamHI/XhoI* to obtain the insert sequence containing the respective number of miRNA copies. Both were ligated according to the standard protocol. All generated plasmids were sequenced to confirm the correct insertion (Eurofins Genomics Europe Sequencing, Germany). All mature miRNA sequences cloned into the expression plasmid were derived from murine miRNAs (mmu‐miRs, here referred to as “miRs”) and listed in the supporting information (Table S1). For initial vector comparison, the miRNASelect pEGP‐miR (pEGP‐miR) plasmid was constructed as described previously [20]. The PCR primers used to amplify premiR sequences including ∼100 base pairs of the up‐ and downstream flanking regions are listed in the supporting information (Table S2).

### 2.4 Transient transfection of miRNA mimics and plasmids

100 nM of the respective miRNA or amiRNA mimic (Horizon Discovery, United Kingdom) were transfected into CHO cells at a concentration of 31,500 vc/100 µL. As a positive control, a functional anti‐FUT8 siRNA (GAACACUCAUCUUGGAAUCUU) [27] was transfected, while a nontargeting siRNA (Qiagen, Germany) (NT) and nontransfected cells (UT) were used as negative controls. For the transfection of all mimics, TransIT‐X2 reagent (Mirus Bio, USA) was used at a concentration of 0.3 µL/well. TransIT‐X2 reagent and RNA were mixed and incubated for 20 min at RT and then added to the cells. Subsequently, the transfected cells were seeded into a 96‐well U‐bottom suspension plate (Greiner Bio‐One, Austria) which was sealed afterwards with a BREATHseal (Greiner Bio‐One, Austria). Cells were cultivated under standard conditions at 600 rpm until further readout. For transient transfection of plasmids, cells were also treated as described here without adding antibiotics.

### 2.5 Stable transfection of miRNA‐/siRNA‐plasmids

Stable transfection of CHO cells was performed using the TransIT‐X2 Dynamic Delivery System. Briefly, 30,000 vc/mL was plated in 2.5 mL medium in 6‐well plates and incubated agitated for 24 h. A mixture of 250 µL medium, 2.5 µg plasmid, and 7.5 µL transfection reagent was then added to the incubated cells. Following transfection, cells were incubated for 24 h staticly at standard conditions and then transferred to shaken conditions for another 24 h until transfection efficiency was determined via fluorescence measurement of the GFP marker gene. Furthermore, 48 h post transfection, 3 µg/mL blasticidin (invivoGen, USA) was added to the medium with each passaging step until the viability of the cells completely recovered. Cells were passaged every 2 to 3 days to 2 × 10^5^ vc/mL and viability and cell concentration were monitored for each passage.

### 2.6 Single cell cloning

As soon as the antibiotic selection of stable cell pools was completed, the stable CHO pool containing pcDNA6.2‐GW/EmGFP‐miR‐3096b‐5p was selected for single cell cloning without further addition of antibiotics to screen for clones with potentially stronger phenotypic effects. A limiting dilution was performed by seeding cells from the cell pool at 0.3 vc/well in a 96‐well F‐bottom suspension plate (Greiner Bio‐One, Austria) sealed with a BREATHseal. After seeding, the plates were assayed using light microscopy to select only wells with singe cells for further experiments. Cells were cultivated staticly using standard conditions until growth was observed and these clones were transferred to 24‐well and subsequently 6‐well U‐bottom suspension plates (Greiner Bio‐One, Austria) and cultivated with agitation until assayed for mAb glycosylation, gene regulation and mAb productivity.

### 2.7 Flow cytometry

The percentage of cells expressing GFP was measured at several checkpoints using the MACSQuant Analyzer 10 (Miltenyi Biotec, Germany). GFP was measured at 525/50 nm using a 488 nm laser (Channel B1) for fluorescence excitation. Subsequent data analysis was performed with the MACSQuantify software.

### 2.8 Quantitative reverse transcription polymerase chain reaction (qPCR)

The total RNA of analyzed cells was isolated using the miRNeasy Mini Kit (Qiagen, Germany) following the manufacturer´s protocol. For target regulation analysis, cDNA was synthesized using the High Capacity cDNA Reverse Transcription kit (Applied Biosystems, USA) according to the manufacturer's protocol. QPCR was performed on a LightCycler 480 instrument (Roche, Switzerland) using CHO‐specific TaqMan assays for FUT8 (Cg04433064_m1, Applied Biosystems, USA) and for fucose kinase (FUK) (Cg04505402_m1). Glyceraldehyde‐3‐phosphate dehydrogenase (GAPDH (Cg04424038_gH)) was used as a housekeeping reference. QPCR was conducted according to the TaqMan Gene Expression Master Mix protocol (Applied Biosystems, USA). For miRNA quantification, cDNA was transcribed using the miRCURY LNA RT Kit (#339340, Qiagen). QPCR was performed with the above described instrument using the miRCURY SYBR‐Green PCR Kit. Specific miRCURY LNA miRNA PCR primers were used for miR‐34a‐5p (YP00204486), miR‐3096b‐5p (custom synthesis based on miRNA sequence), and mmu‐U6‐snRNA (YP02119464), respectively, following the manufacturer´s protocol. Data were evaluated using the 2^−ΔΔct^ method [68]. For transient experiments, cells were analyzed 48 h post transfection while stably transfected cells were analyzed after the selection process was completed.

### 2.9 Protein A chromatography

To determine the antibody concentration in samples of supernatants miRNA‐regulated CHO cell cultures, protein A high‐performance liquid chromatography (HPLC) was performed. Supernatants were 0.2 µm filtered (AcroPrep Advance 96 Filter Plates, Pall Corporation, USA) and assayed on an UltiMate 3000 system (Thermo Fisher Scientific Inc., USA) with a Protein A POROS A 20 µm column (Thermo Fisher Scientific Inc.) A phosphate buffer was used for equilibration (pH 7.5) and for elution (pH 2.5). The mAb was detected by UV absorbance at 280 nm. Concentration was calculated by comparison to a dilution series of a reference mAb standard that was measured the same way and productivity was referenced to the benchmark cell line.

### 2.10 Glycosylation analysis of intact antibody

Glycosylation analysis was performed by analyzing the intact mAb on a DionexUltiMate 3000 LC coupled to an Orbitrap Fusion Lumos mass spectrometer (LC‐MS). Protein A purified cell supernatant was analyzed using a Thermo Scientific MAbPac RP guard‐ and separation column (2.1 × 10 and 1 × 100 mm) thermostated at 80°C. The LC‐MS method followed our recently published method [69] with slight modifications [37]. Charge‐deconvolution, peak assignment, and annotation of the generated mass spectrometric data were performed with Intact Mass (Protein Metrics, USA). The m/z range for the charge deconvolution was 2600–3400. Differences in the glycosylation patterns were evaluated by comparing the intensities of the assigned glycoforms on the respective mAbs to the glycoforms of the NT or mock control. Fold changes were calculated by summation of the value of the relevant glycoforms (G0F/G1F, G1F/G1F (G0F/G2F), G1F/G2F) of the sample with subsequent division by the respective reference value. Example spectra are presented in the supporting information for all described cell pools (Figure S1). Apart from fucosylation, the degree of galactosylation and sialylation of the produced mAbs can be found in the supporting information for all described cell pools (Table S3).

### 2.11 Metabolite analysis

Washed and shock‐frozen (−80°C) cell pellets of stable CHO pools containing the pcDNA6.2‐GW/EmGFP‐miR plasmid harboring the mock sequence, a FUT8 siRNA, miR‐3096b‐5p, 2 x miR‐3096b‐5p, miR‐34a‐5p, or 2 x miR‐34a‐5p were used for metabolite analysis. The analytical workflow followed our recently published method with slight modifications [70]. Briefly, the method included a cell extraction step and a HILIC‐MS analysis step of the cell extracts, followed by statistical data evaluation. Detailed sample preparation, the internal standard used for sample preparation, and the used metabolite standard are given in the supporting information (Supplementary information 1). The standard was used for the determination of the retention times of the target metabolites. For the evaluation, ion traces of the target analytes were extracted and the peaks were integrated. For the execution of the principal component analysis (PCA), the raw data were normalized to the applied cell number. Further data pretreatment steps comprised centering, scaling (Pareto scaling), and transformation (power transformation) [71].

### 2.12 Statistical analysis

Data are presented as mean ± SD. The GraphPad Prism software (Version 8.0.1) (GraphPad Software, San Diego, CA, USA) was used for statistical analysis. Statistical significance of data was tested by ordinary one‐way ANOVA with Dunnett's correction. A *p*‐value < 0.05 was considered significant. The PCA was created with JMP 17.0.0 (SAS, Cary, NC, USA).

## 3 RESULTS

### 3.1 Evaluation of stable miRNA expression systems

**FIGURE 1:**
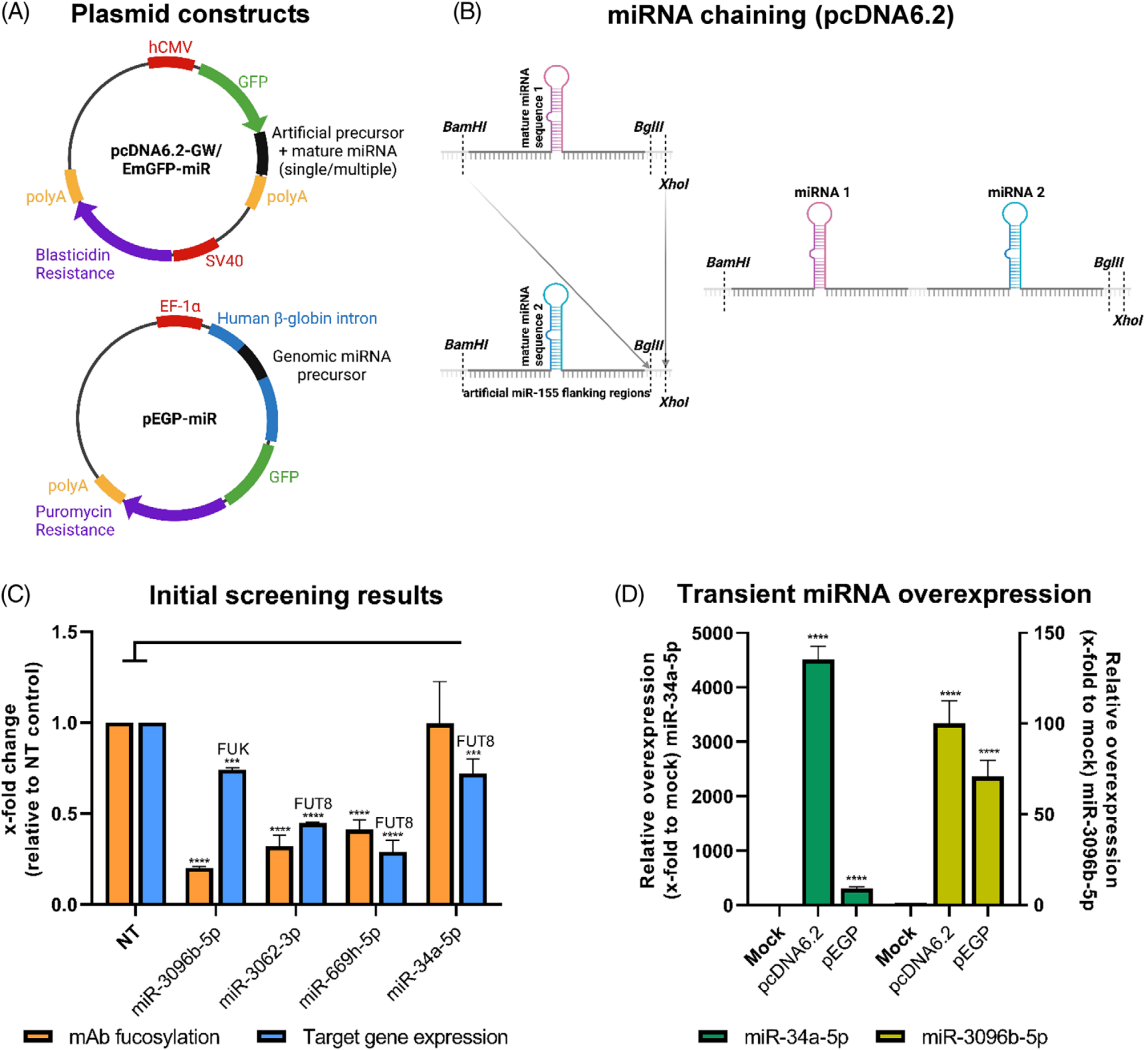
Plasmid system comparison for stable microRNA (miRNA) overexpression in Chinese hamster ovary (CHO) cells. (A) Schematic representation of the tested plasmid systems. The pcDNA6.2‐GW/EmGFP‐miR plasmid harbors a mature miRNA sequence of choice in miR‐155 flanking regions, which are located in the 3′untranslated region (3′UTR) of the green fluorescence protein (GFP). The pEGP‐miR plasmid incorporates a miRNA amplified with its genomic context from a reference genome into a human β‐globin intron in front of a GFP reporter. Created with BioRender.com. (B) Simplified schematic overview of the pcDNA6.2‐GW/EmGFP‐miR cloning system allowing chaining of multiple miRNAs of the same or different origin. A combination of *BglII/XhoI* restriction sites is used to obtain the backbone and *BamHI/XhoI* to obtain the insert sequence. Created with BioRender.com. (C) Previous results from our work [37] were used as a starting point for stable miRNA expression. Relative quantification of target mRNA expression (blue) 48 h after transient miRNA mimic transfection in comparison to nontargeting control siRNA (NT) transfection by quantitative reverse transcription polymerase chain reaction (qPCR) analysis. Analysis of mAb fucosylation in the supernatant (orange) 72 h after the same transfection compared to NT. Target genes are fucosyltransferase 8 (FUT8) for miR‐3062‐3p, miR‐669h‐5p and miR‐34a‐5p as well as fucokinase (FUK) for miR‐3096b‐5p. QPCR data are presented as calculated x‐fold change normalized to glyceraldehyde‐3‐phosphate dehydrogenase (GAPDH) housekeeping control and relative to NT (*n* = 3 biological replicates, mean ± SD). Data for mAb fucosylation is presented as calculated normalized relative shares for every glycoform (*n* = 3 biological replicates, mean + SD). (D) Comparison of mature miRNA overexpression after transient plasmid transfection of pcDNA6.2‐GW/EmGFP‐miR‐3096b‐5p, pcDNA6.2‐GW/EmGFP‐miR‐34a‐5p, pEGP‐miR‐3096b‐5p and pEGP‐miR‐34a‐5p into mAb producing CHO cells. MiRNA overexpression was assayed 48 h after transfection via qPCR. QPCR data are presented as calculated x‐fold change normalized to mmu‐U6‐snRNA housekeeping control and relative to mock (*n* = 3 biological replicates, mean ± SD). Significance was tested by ordinary one‐way ANOVA with Tukey's multiple comparisons test (**** = *p* ≤ 0.0001; ***: *p* ≤ 0.001; **: *p* ≤ 0.01; *: *p* ≤ 0.05; ns: *p* > 0.05).

Although miRNAs have previously been described as efficient engineering tools, their effect on glycosylation remained largely unexplored. In the current study, we aimed at developing stable miRNA‐based tools for glyco‐engineering which enable tailor‐made effect modulation, for example, the production of biosimilars. In CHO DG44 cell lines, efficient miRNA overexpression is dependent on the plasmid system [23], therefore we initially evaluated two plasmids for stable overexpression of miRNAs in CHO‐K1 production cells to facilitate efficient control of the model readout mAb fucosylation (Figure 1A). While the pcDNA6.2‐GW/EmGFP‐miR plasmid allows cloning of a mature miRNA sequence of choice, this system also enables the expression of any miRNA sequence of choice and cloning of multiple copies of miRNA inserts (Figure 1B). In contrast, the second plasmid system pEGP‐miR allows cloning of a native miRNA including its genomic context, that is, the native flanking regions of the respective miRNA into the human β‐globin intron in front of GFP. However, the latter system is limited as it allows only cloning of one copy of the miRNA of choice and, additionally, a genomic template of the miRNA must be available.

Our recently published work identified several miRNAs, which after transient transfection decreased mAb fucosylation in CHO cells by regulating key enzymes in the fucosylation pathway [37]. A set of candidate miRNAs, which all reduced the respective target gene expression and protein fucosylation after transient transfection into CHO production cells, was selected (Figure 1C). For the initial evaluation of expression systems, miR‐34a‐5p targeting FUT8 (0.99‐fold fucosylation and 0.72‐fold target regulation) and miR‐3096b‐5p targeting FUK (0.2‐fold fucosylation and 0.74‐fold target regulation) were cloned as single copies into both expression plasmid systems to cover one strong and one weak fucosylation regulator and two different target enzymes. Additionally, two miRNAs targeting FUT8, miR‐3062‐3p (0.32‐fold fucosylation and 0.44‐fold target regulation) and miR‐669h‐5p (0.41‐fold fucosylation and 0.29‐fold target regulation), were used for later validation experiments. A CHO‐K1 cell line producing an IgG1‐type mAb obtained from Rentschler Biopharma [67] was transiently transfected with the resulting pcDNA6.2‐GW/EmGFP‐miR‐34a‐5p and 6.2‐GW/EmGFP‐miR‐3096b‐5p plasmids or the pEGP‐miR‐34a‐5p and pEGP‐miR‐3096b‐5p plasmids. Cells transfected with the respective plasmids carrying a nontargeting miRNA sequence served as reference (mock). MiRNA overexpression in CHO cells was analyzed 48 h after transfection via qPCR and revealed a significantly higher miRNA expression with pcDNA6.2‐GW/EmGFP‐miR (4513‐fold for miR‐34a‐5p and 100‐fold for miR3096b‐5p) than with pEGP‐miR (306‐fold for miR‐34a‐5p and 70‐fold for miR‐3096b‐5p) for both miRNAs (Figure 1D). Therefore, the pcDNA6.2‐GW/EmGFP‐miR plasmid was used for all further experiments and for cloning of miR‐3062‐3p and miR‐669h‐5p to maximize miRNA overexpression.

### 3.2 Comparison of transient and stable miRNA overexpression systems

**FIGURE 2:**
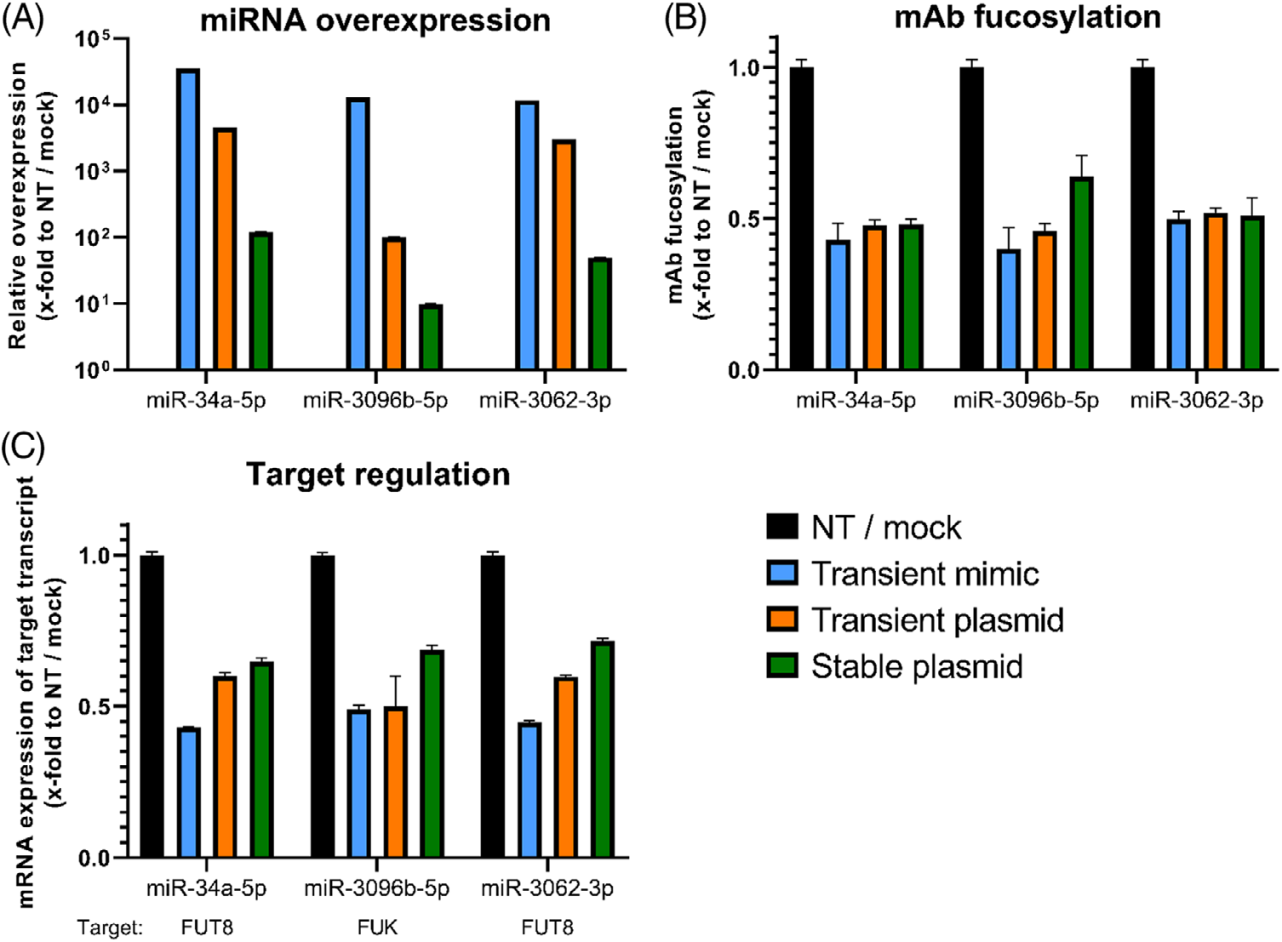
Comparison of transient miRNA mimic transfection, transient plasmid transfection, and stable plasmid transfection in Chinese hamster ovary (CHO) cells for the three example miRNAs miR‐34a‐5p, miR‐3096b‐5p and miR‐3062‐3p. For all plasmid‐based transfections, the pcDNA6.2‐GW/EmGFP‐miR system was used. (A) Overexpression of mature miRNA. Transient miRNA mimic and plasmid transfection were assayed 48 h after transfection, stable plasmid transfection after cells completed antibiotic selection. Quantitative reverse transcription polymerase chain reaction (qPCR) data are presented as calculated x‐fold change normalized to mmu‐U6‐snRNA housekeeping control and relative to nontargeting control siRNA (NT) for miRNA mimics or mock for plasmid transfections (*n* = 3 biological replicates, mean ± SD). (B) Analysis of monoclonal antibody (mAb) fucosylation in the supernatant compared to NT or mock. Data for mAb fucosylation is presented as calculated normalized relative shares for every glycoform (*n* = 3 biological replicates, mean + SD). (C) Target regulation after transient transfection of miRNA mimics and plasmids or stable plasmid transfection assayed 48 h after transfection, or as soon as cells got stable. Target genes were fucosyltransferase 8 (FUT8) for miR‐34a‐5p and miR‐3062‐3p or fucokinase (FUK) for miR‐3096b‐5p. QPCR data are presented as calculated x‐fold change normalized to glyceraldehyde‐3‐phosphate dehydrogenase (GAPDH) housekeeping control and relative to NT or mock (*n* = 3 biological replicates, mean + SD). Significance was tested by ordinary one‐way ANOVA with Tukey's multiple comparisons test (**** = *p* ≤ 0.0001; ***: *p* ≤ 0.001; **: *p* ≤ 0.01; *: *p* ≤ 0.05; ns: *p* > 0.05).

As stable overexpression of modulatory miRNAs is mandatory for a potential industrial application, effects generated by transient transfection of miRNAs were compared to those obtained by stable overexpression of these miRNAs. Therefore, miRNA overexpression levels, target regulation, and phenotypic changes in mAb fucosylation were analyzed for transient miRNA mimic transfection, transient plasmid transfection, as well as stable plasmid transfection. Cells were transiently transfected with miR‐34a‐5p, miR‐3096b‐5p, miR‐3062‐3p mimics or pcDNA6.2‐GW/EmGFP‐miR containing a single copy of either of these miRNAs. Additionally, the same plasmid transfection was performed with subsequent antibiotic selection to generate stable CHO cell pools. GFP expression and mAb fucosylation were monitored for selected cell pools every 2–3 days to ensure stable integration of the plasmid (Figure 2A–C). Mimic‐based transient transfections showed consistently higher expression levels for mature miRNAs miR‐34a‐5p (∼36,000‐fold), miR‐3096b‐5p (∼13,000‐fold) and miR‐3062‐3p (∼12,000‐fold), followed by transient plasmid‐based transfections with 4513‐fold for miR‐34a‐5p, 100‐fold for miR‐3096b‐5p and 3005‐fold for miR‐3062‐3p, compared to mock pools. The stable miRNA expressing cell pools reached considerably lower expression levels for these miRNAs by 120‐fold (miR‐34a‐5p), 10‐fold (miR‐3096b‐5p) and 50‐fold (miR‐3062‐3p) (Figure 2A). The phenotypic effect of afucosylation of the secreted mAb was evaluated via mass spectrometry (Figure 2B). While cells transfected with miR‐34a‐5p and miR‐3062‐3p reached the same low degree of fucosylation (∼0.5‐fold), independent of the mode of transfection, cells transfected with miR‐3096b‐5p displayed a significant loss of effect for the stable plasmid‐based approach (stable plasmid fucosylation 0.64‐fold, transient mimic fucosylation 0.40‐fold). Analysis of the down‐regulation of the respective target genes for the miRNAs miR‐34a‐5p, miR‐3062‐3p, and miR‐3096b‐5p revealed the weakest regulation of their respective targets FUT8 and FUK (0.71‐fold to 0.64‐fold) for the stable cell pools, followed by the transient plasmid transfections (0.59‐fold to 0.50‐fold) and the transient mimic transfections (0.49‐fold to 0.43‐fold) (Figure 2C). As a discrepancy in afucosylation between transiently transfected and stable CHO cell pools was observed for miR‐3096b‐5p, this miRNA was chosen as an adequate model for expression optimization, using mAb afucosylation as a relevant readout for biosimilar production.

### 3.3 Single cell cloning only yields a few slightly improved clones

**FIGURE 3:**
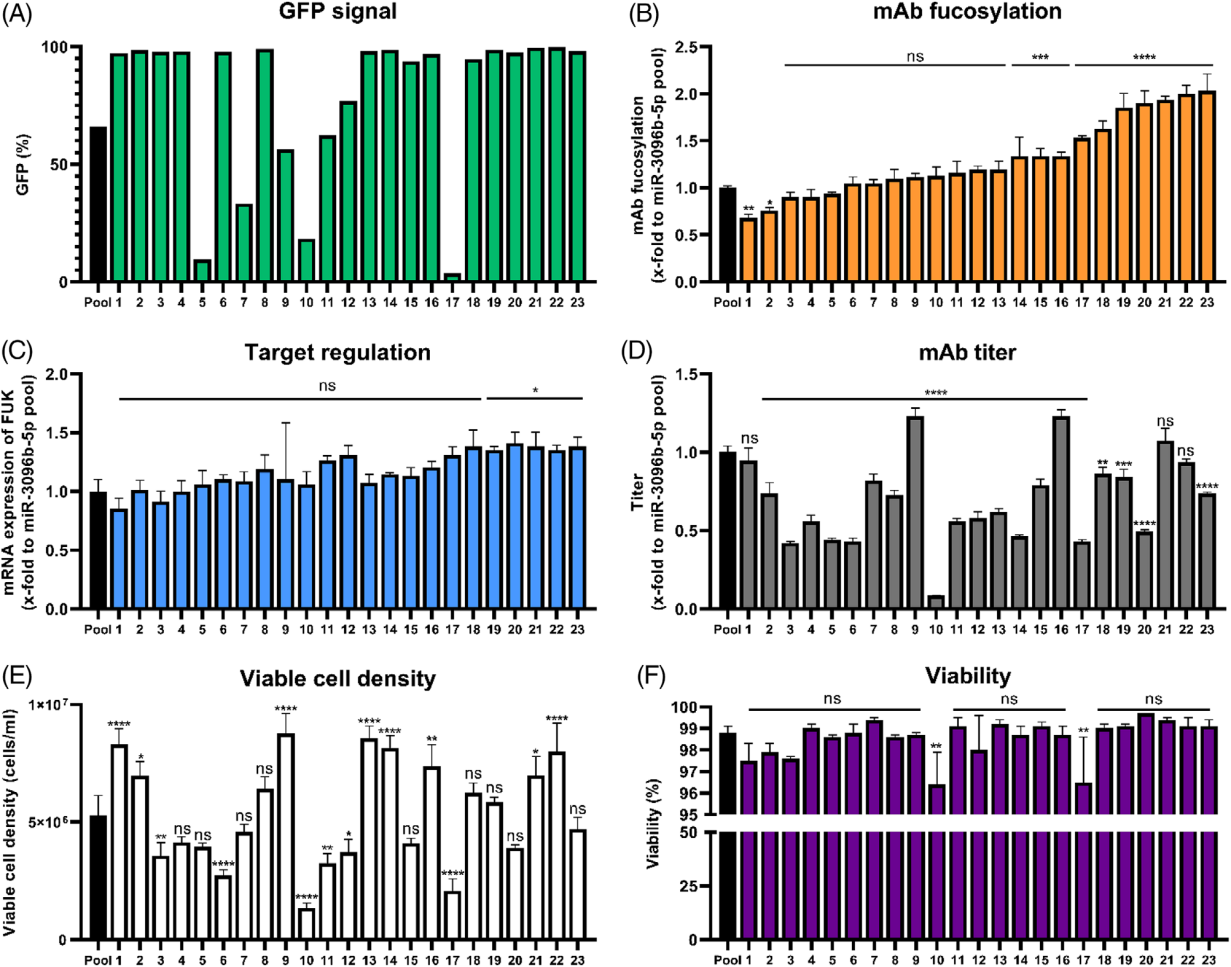
Single cell cloning of stable miR‐3096b‐5p pool cloned in the pcDNA6.2‐GW/EmGFP‐miR. (A) Comparison of percentage of cells exhibiting green fluorescence protein (GFP) signal of 23 clonal cell lines to the cell pool assayed via flow cytometry. (B) Fucosylation of secreted monoclonal antibody (mAb). Data for mAb fucosylation are presented as calculated normalized relative shares for every glycoform and relative to the miR‐3096b‐5p expressing cell pool (*n* = 3 biological replicates, mean + SD). (C) Gene regulation of fucokinase (FUK) by miR‐3096b‐5p in all 23 clonal cell lines. Quantitative reverse transcription polymerase chain reaction (qPCR) data are presented as calculated x‐fold change normalized to glyceraldehyde‐3‐phosphate dehydrogenase (GAPDH) housekeeping control and relative to the miR‐3096b‐5p expressing cell pool (*n* = 3 biological replicates, mean + SD). (D) Titer analysis of single cell clones assayed via protein A chromatography in a 6‐day batch experiment relative to the miR‐3096b‐5p expressing cell pool (*n* = 3 biological replicates, mean + SD). (E) Viable cell density measured via trypan blue exclusion in a 6‐day batch experiment relative to the miR‐3096b‐5p expressing cell pool (*n* = 3 biological replicates, mean + SD). (F) Measurement of viability via trypan blue exclusion in a 6‐day batch experiment relative to the miR‐3096b‐5p expressing cell pool (*n* = 3 biological replicates, mean + SD). Significance was tested by ordinary one‐way ANOVA with Tukey's multiple comparisons test (**** = *p* ≤ 0.0001; ***: *p* ≤ 0.001; **: *p* ≤ 0.01; *: *p* ≤ 0.05; ns: *p* > 0.05).

One of the currently used standard methods in industrial cell line development is single cell cloning, which should minimize genetic heterogeneity in the cell population and provide the opportunity to select high expressing cell lines with the most desirable phenotype. Therefore, we evaluated whether single cell cloning of the stable miRNA expressing cell pool of our model miR‐3096b‐5p would enable the generation of cell clones with higher mAb afucosylation capacity, compared to the established cell pools. Single cell cloning resulted in 23 clonal cell lines (Figure 3). GFP expression showed broad distributions with a majority of 16 cell lines consisting of cell populations with a very high percentage (close to 100%) of GFP expressing cells (Figure 3A), indicating a high degree of stable and active integration of the plasmid harboring the miRNA. However, only cell lines 1 and 2 showed significantly lowered degrees of fucosylation compared to the cell pool with 0.67‐fold and 0.75‐fold and an overexpression of mature miRNA similar to the pool with ∼10‐fold (Figure S3A), respectively. The majority of cell lines expressing this miRNA displayed similar or even increased degrees of fucosylation (Figure 3B). A trend towards a correlation between target repression and the degree of fucosylation was observed for single cell clones, but no cell clone with significantly stronger FUK regulation was identified when compared to the cell pool (Figure 3C). Regarding process performance, productivity was overall negatively influenced by single cell cloning, as most clones except clone 9 and 16 showed lower titers compared to the pool in a 6‐day batch experiment (<1‐fold) (Figure 3D). Many clones displayed poor growth (Figure 3E), while viability was generally not affected (Figure 3F). Overall, single cell cloning of our model miR‐3096b‐5p did not provide a suitable option to reliably improve the cellular phenotype.

### 3.4 Chaining of miRNAs reliably increased effect strength

**FIGURE 4:**
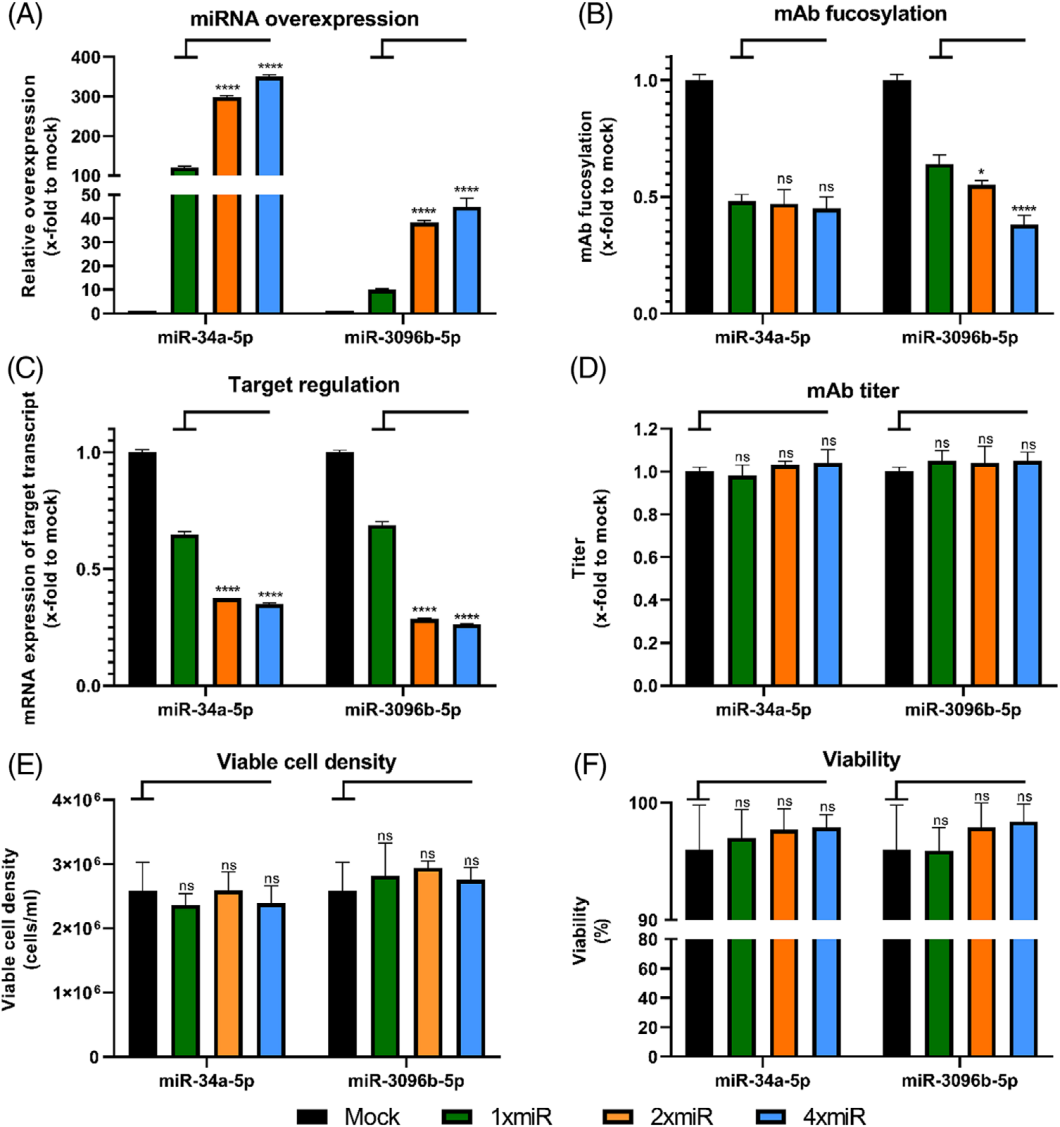
Chaining of multiple miRNA copies using the pcDNA6.2‐GW/EmGFP‐miR‐34a‐5p or pcDNA6.2‐GW/EmGFP‐miR‐3096b‐5p plasmid to increase stably induced effects. Plasmids harboring 1, 2, or 4 copies of the respective miRNA were tested. (A) Level of mature miRNA in stably overexpressing Chinese hamster ovary (CHO) cell pools. QPCR data are presented as calculated x‐fold change normalized to mmu‐U6‐snRNA housekeeping control and relative to mock (*n* = 3 biological replicates, mean + SD). (B) Analysis of fucosylation on the secreted monoclonal antibody (mAb) via mass spectrometry. Data are presented as calculated normalized relative shares for every glycoform and relative to mock (*n* = 3 biological replicates, mean + SD). (C) Regulation of target genes fucosyltransferase 8 (FUT8) for miR‐34a‐5p or fucokinase (FUK) for miR‐3096b‐5p expressing cell pools assayed via qPCR. Data are presented as calculated x‐fold change normalized to glyceraldehyde‐3‐phosphate dehydrogenase (GAPDH) housekeeping control and relative to mock (*n* = 3 biological replicates, mean + SD). (D) Titer analysis of single and chained copy plasmids assayed via protein A chromatography in a 6‐day batch experiment relative to mock (*n* = 3 biological replicates, mean + SD). (E) Viable cell density was assayed via trypan blue exclusion in a 6‐day batch experiment and calculated relative to mock (*n* = 3 biological replicates, mean + SD). (F) Viability measurement via trypan blue exclusion in a 6‐day batch experiment relative to mock (*n* = 3 biological replicates, mean + SD). Significance was tested by ordinary one‐way ANOVA with Tukey's multiple comparisons test (**** = *p* ≤ 0.0001; ***: *p* ≤ 0.001; **: *p* ≤ 0.01; *: *p* ≤ 0.05; ns: *p* > 0.05).

It was previously shown that chaining of miRNAs can improve product titer [6, 19] and miRNA overexpression [23]. Therefore, we evaluated the option to chain several miRNAs within the pcDNA6.2‐GW/EmGFP‐miR system to increase mAb afucosylation. To achieve this, the pcDNA6.2‐GW/EmGFP‐miR plasmids containing the model miR‐3096b‐5p and the reference miR‐34a‐5p were further modified to contain two or four copies of the respective miRNA per plasmid (Figure 1B). After stable integration into CHO production cells, miRNA overexpression, target gene regulation, and mAB fucosylation were determined and compared to the values obtained with the respective stable cell pool containing single copy plasmids. For both miRNAs a significant increase in overexpression was observable when introducing multiple copies of miRNAs (Figure 4A). Stable overexpression of miR‐34a‐5p could be increased from 120‐fold (single copy) to 298‐fold (two copies) and 350‐fold (four copies). Regarding miR‐3096b‐5p, the resulting overexpression could be elevated from 10‐fold (single copy) to 38‐fold (two copies) and 45‐fold (four copies). All copy numbers of miR‐34a‐5p resulted in the same degree of fucosylation with ∼0.5‐fold (Figure 4B). In contrast, the stable cell pools containing miR‐3096b‐5p secreted mABs with fucosylation levels correlating to the overexpression and target regulation with 0.64‐fold (single copy) to 0.55‐fold (two copies) and 0.38‐fold (four copies). Both miRNAs showed a stronger target regulation capacity when more than one miRNA copy was used (Figure 4C). For miR‐34a‐5p, regulation of its target FUT8 could be improved from 0.64‐fold (single copy) to 0.37‐fold (two copies) and 0.35‐fold (four copies). Stable expression of the plasmid containing miR‐3096b‐5p, regulating FUK, also led to an enhanced regulation when multiple copies of the miRNA were integrated, from 0.68‐fold (single copy) to 0.28‐fold (two copies) and 0.26‐fold (four copies). For all investigated copy numbers of both miRNAs, none of the analyzed process parameters was negatively influenced, as mAB titer (Figure 4D), viable cell concentration (Figure 4E), and viability (Figure 4F) were not significantly altered during a 6‐day batch culture.

### 3.5 AmiRNA implementation as improved engineering tool

**FIGURE 5:**
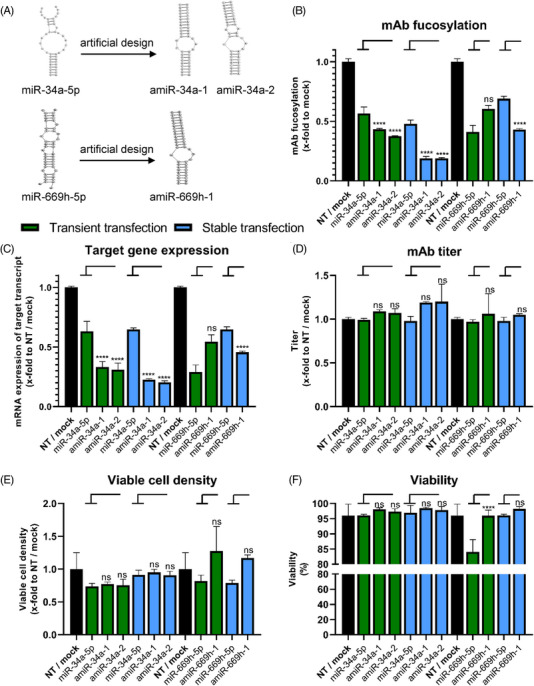
Implementation of stable artificial miRNA (amiR/amiRNA) expression in Chinese hamster ovary (CHO) cells using the pcDNA6.2‐GW/EmGFP‐miR plasmid system. AmiRNAs for stable expression were chosen based on our recent results and data presented in green is based on these transient results [37] (A) Schematic representation of the (a)miRNA‐mRNA duplex for miR‐34a‐5p, amiR‐34a‐1, amiR‐34a‐2, miR‐669h‐5p and amiR‐669h‐1 and fucosyltransferase 8 (FUT8) from our previous results and their respective regulation on the fucosylation of a secreted monoclonal antibody (mAb) after transient transfection. Fucosylation data are presented as calculated normalized relative shares for every glycoform and relative to nontargeting control siRNA (NT) (*n* = 3 biological replicates, mean + SD). (B) Regulation of mAb fucosylation after transient mimic and stable plasmid transfection of amiRNAs and their native counterparts. Transient data were determined 72 h post transfection and stable data, as cell pools completed the antibiotic selection process. Data are presented as calculated normalized relative shares for every glycoform and relative to NT for mimic transfections or mock for stable integration (*n* = 3 biological replicates, mean + SD). (C) Regulation of the target genes fucosyltransferase 8 (FUT8) for miR‐34a‐5p, miR‐669h‐5p, and their artificial variants (amiRs) expressing cell pools and transient transfections of respective miRNA mimics assayed via quantitative reverse transcription polymerase chain reaction (qPCR). Data are presented as calculated x‐fold change normalized to glyceraldehyde‐3‐phosphate dehydrogenase (GAPDH) housekeeping control and relative to mock (*n* = 3 biological replicates, mean + SD). (D) Titer analysis of transiently transfected amiRNA mimics and their native counterparts 72 h post transfection and stable plasmid based integration in a 6‐day batch experiment. MAb Titer was measured via protein A chromatography relative to NT or mock (*n* = 3 biological replicates, mean + SD). (E) Viable cell density determined via trypan blue exclusion. For transient mimic transfection, cell density was determined 72 h post transfection, stable cell lines were assayed after a 6‐day batch experiment. X‐fold changes are calculated relative to NT or mock (*n* = 3 biological replicates, mean + SD). (F) Viability of CHO cells 72 h post transient transfection with amiRNAs and their native counterparts and during a 6‐day batch experiment of stably expressing cell pools. Viability is calculated relative to NT or mock (*n* = 3 biological replicates, mean + SD). Significance was tested by ordinary one‐way ANOVA with Tukey's multiple comparisons test (**** = *p* ≤ 0.0001; ***: *p* ≤ 0.001; **: *p* ≤ 0.01; *: *p* ≤ 0.05; ns: *p* > 0.05).

In addition to single cell cloning and chaining of miRNAs, which aimed towards an enhancement of native miRNA expression, we explored a third approach to implement miRNAs as engineering tools using artificial molecules. AmiRNAs, which we recently described as improved variants of their native counterparts in transient experiments, were here tested for their suitability for stable integration (Figure 5A) [37]. Therefore, best regulating amiRs of the previously described miR‐34a‐5p (Figure 5B–F) and miR‐669h‐5p (Figure 5B–F), were cloned into the expression plasmid pcDNA6.2‐GW/EmGFP‐miR and stably integrated into CHO production cells. AmiR variants of miR‐3096b‐5p were not chosen due to metabolic shifts observed in our previous work. Subsequently, the resulting mAb fucosylation was determined and compared to the fucosylation level obtained in transient transfections of the respective mimics (Figure 5B). The two artificial variants amiR‐34a‐1 and amiR‐34a‐2 induced strong afucosylation after transient transfection (0.43‐fold and 0.37‐fold remaining fucosylation, respectively). However, when integrated stably into the genome, an even lower degree of fucosylation (0.19‐fold for both) was observed. While low fucosylation was obtained with the second miRNA miR‐669h‐5p after transient transfection (0.41‐fold), stable integration of this miRNA caused an increase in fucosylation (0.69‐fold). Its artificial variant lost afucosylation capacity to some degree (0.60‐fold) after transient transfection. However, after stable transfection of this amiRNA, strongly decreased fucosylation (0.43‐fold) was observed. All miRNAs and their amiRNA variants target FUT8 and regulation analysis via qPCR revealed a downregulation correlating strongly with the degree of fucosylation (Figure 5C). For all cell pools with stably integrated amiRNAs, analysis of mAb titers obtained in a 6‐day batch showed no adverse effects on productivity. The two stably integrated amiR variants of miR‐34a‐5p even increased the titer significantly to 1.2‐fold (Figure 5D). Viable cell concentration (Figure 5E) and viability (Figure 5F) were also not negatively influenced by stable integration of the miR‐34a‐5p amiRs. However, transient experiments with native miR‐669h‐5p resulted in poorly growing cells coupled with an adverse effect of lowered cellular viability [37]. Its artificial variant amiR‐669h‐5p‐1 was designed to abolish the adverse effect of decreased viability, which was observed in transient experiments. After stable integration of this amiRNA, normal cell growth and viability coupled with low fucosylation were achieved, demonstrating the potential of amiRNAs to eliminate unfavorable side effects associated with the respective natural miRNAs.

### 3.6 Metabolite analysis of stable cell pools

**FIGURE 6:**
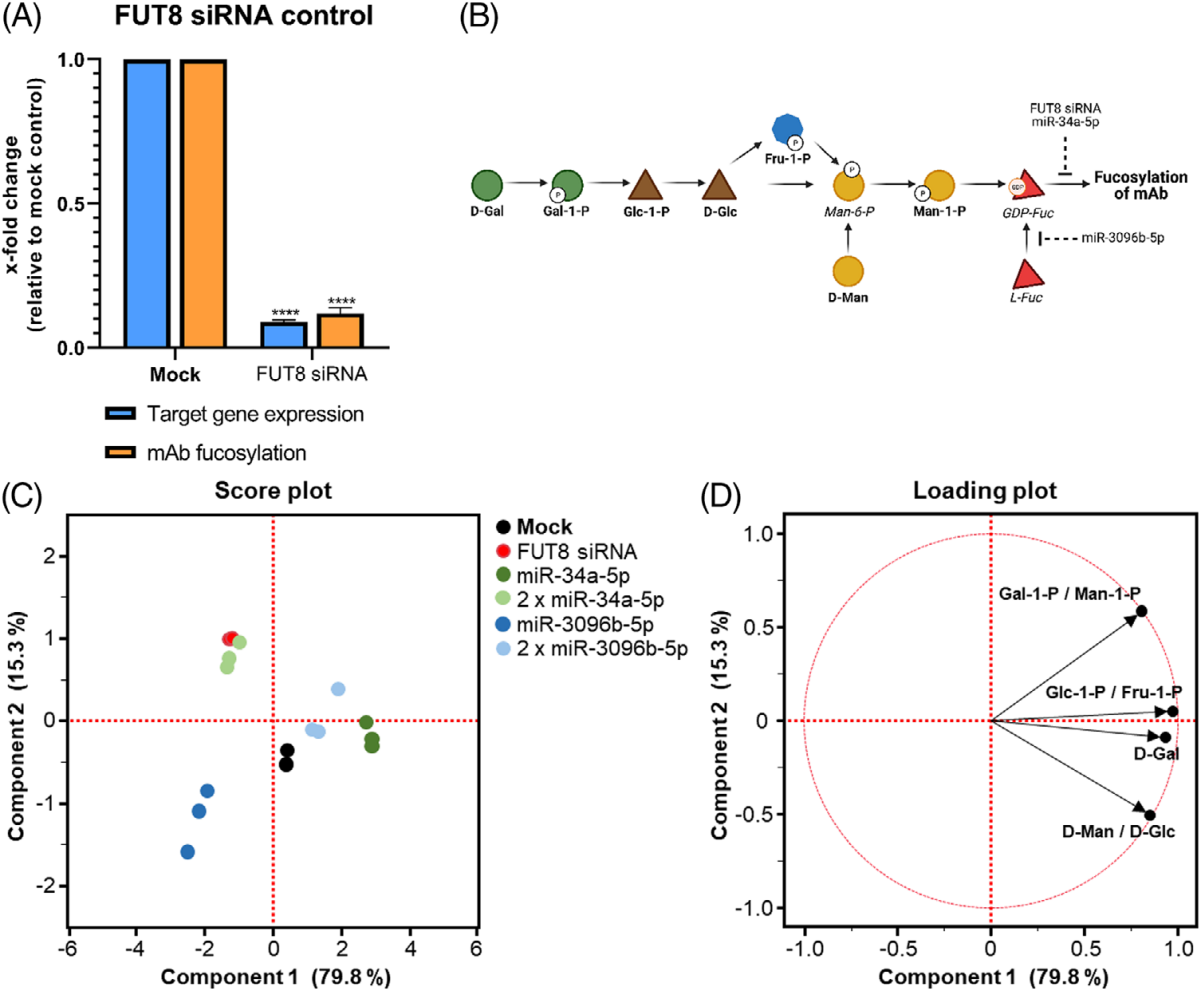
Metabolite analysis of selected stable cell pools. (A) Establishment of a positive control for metabolite analysis. A fucosyltransferase 8 targeting small interfering RNA (FUT8 siRNA) was stably expressed in Chinese hamster ovary (CHO) production cells. Fucosylation on the secreted monoclonal antibody (mAb) was measured via mass spectrometry. Data are presented as calculated normalized relative shares for every glycoform and relative to mock (*n* = 3 biological replicates, mean + SD). Regulation of the target gene FUT8 was assayed via quantitative reverse transcription polymerase chain reaction (qPCR). Data is presented as calculated x‐fold change normalized to glyceraldehyde‐3‐phosphate dehydrogenase (GAPDH) housekeeping control and relative to mock (*n* = 3 biological replicates, mean + SD). Significance was tested by ordinary one‐way ANOVA with Tukey's multiple comparisons test (**** = *p* ≤ 0.0001; ***: *p* ≤ 0.001; **: *p* ≤ 0.01; *: *p* ≤ 0.05; ns: *p* > 0.05). (B) Simplified representation of the interconversion of the analysed sugars and sugar phosphates in CHO production cells leading to mAb fucosylation. Bold names are metabolites, which are represented in the principal component analysis (PCA), italic names are metabolites shown for schematic overview that were not assayed in the metabolite analysis. Metabolites are as follows: D‐galactose (D‐Gal), galactose‐1‐phosphate (Gal‐1‐P), glucose‐1‐phosphate (Glc‐1‐P), D‐glucose (D‐Glc), fructose‐1‐phosphate (Fru‐1‐P), mannose‐6‐phosphate (Man‐6‐P), D‐mannose (D‐Man), mannose‐1‐phosphate (Man‐1‐P), GDP‐fucose (GDP‐Fuc). Created with BioRender.com. (C) Score plot of the PCA from the metabolite analysis in stably expressing CHO cell pools. (D) Loading plot from the PCA with the loading of the selected metabolites.

In order to better understand the effects of miRNA overexpression on the metabolic state of the cells, we analyzed the levels of metabolic precursor molecules involved in fucosylation in selected cell pools expressing single and chained miRNAs. Therefore, a small‐scale metabolite analysis of selected sugar precursors was conducted. In addition to the mock control containing a plasmid with a nontargeting miRNA, a positive control was introduced in this experiment by cloning a published FUT8 siRNA [27] into the plasmid, followed by stable integration into CHO cells. This positive control showed a strong afucosylation effect (0.12‐fold remaining fucosylation) on the mAb and a very strong regulatory effect on FUT8 (0.09‐fold gene expression) compared to the mock (Figure 6A). The metabolic level of the hexose phosphates glucose‐1‐phosphate (Glc‐1‐P), fructose‐1‐phosphate (Fru‐1‐P), galactose‐1‐phosphate (Gal‐1‐P), mannose‐1‐phosphate (Man‐1‐P) and hexose D‐mannose (D‐Man), D‐glucose (D‐Glc), and D‐galactose (D‐Gal) were chosen as relevant metabolites (Figure 6B). A PCA was performed with the data obtained for the described metabolites. 95.1% of the variation could be explained by PC1 and PC2 (Figure 6C,D). On PC1, the CHO cell pool expressing 2 x miR‐34a‐5p appeared clustered to FUT8 siRNA expressing cells (Figure 6C) in the score plot. Cells expressing 2 x miR‐3096b‐5p or miR‐34a‐5p clustered closely with the mock control. MiR‐3096b‐5p expressing CHO pools clustered with none of the pools mentioned above. PC2 could resolve differences between the cell pools containing single or chained miRNA plasmids by a shift towards the positive area of PC2 for chained plasmids (Figure 6C). It appeared furthermore, that PC2 explained the degree of phosphorylation for the selected metabolites, as nonphosphorylated sugars were found in the negative area and phosphorylated in the positive area of the loading plot (Figure 6D). The cells expressing the chained 2 x miR‐34a‐5p plasmid or FUT8 siRNA showed a higher loading on Gal‐1‐P/Man‐1‐P and miR‐3096b‐5p expressing cells showed a higher loading on D‐Gal and D‐Man/D‐Glc (Figure 6D).

## 4 DISCUSSION

The initial comparison of the two commercially available plasmid systems enabled us to identify pcDNA6.2‐GW/EmGFP‐miR as the more suitable system for stable miRNA overexpression. Different expression levels of miRNAs from plasmids containing a spacer between the promoter and the miRNA were already observed previously. Consequently, using GFP as a rather long spacer sequence similar to the pcDNA6.2 plasmid, the strongest effect of miRNA‐mediated regulation was observed [60]. Differences between transiently transfected miRNA mimics and plasmid based stable miRNA expression were observed in previous studies for readouts like miRNA overexpression [23] or productivity [19, 20]. Previous studies also showed a random distribution of phenotypes (productivity, product quality, growth) when subcloning engineered CHO cell lines [72, 73] or nonproducing cell lines [74, 75] due to the plasticity of the CHO genome, similar to the heterogeneous results we obtained in single cell cloning. As single cell cloning did not eliminate population heterogeneity, the possibility to integrate more miRNA copies per plasmid by chaining thereby increasing the mean effect in the stable pool was evaluated. It appeared that miRNA chains longer than one miRNA trigger a strong increase in overexpression in CHO cells similar to earlier findings [23], target regulation [61], or resulting phenotypic effects like productivity [6, 19] compared to single miRNAs. Naturally, miRNAs can occur in functional clusters either with their own promoter or in an intron using the promoter of the respective gene [76]. Apart from clustering of different miRNAs, duplication of the same miRNA is a major reason for creating efficient and evolutionarily conserved miRNA clusters, similar to the chained miRNAs presented here [77, 78, 79]. In addition to chaining multiple miRNA copies, the applicability of amiRNAs to modulate fucosylation without inducing adverse side effects was shown. Using amiRNA variants of miR‐34a‐5p, which is a known FUT8 regulator in human tumour cells [80, 81], we could adjust the stable afucosylation effect of the native miRNA. Our results with stable expression of amiRNAs also demonstrate the option to make otherwise unfavourable miRNAs usable for bioprocessing, as the natural variant of miR‐669h‐5p decreased viability in transient experiments [37], while our artificial variant of this miRNA did not show this detrimental side effect. Lastly, our study aimed to investigate possible changes in metabolite levels as a response to miRNA‐mediated regulation in a small‐scale metabolome analysis. Generally, all tested cell lines showed a very similar profile of the tested metabolites, leading to the conclusion that the stably overexpressed miRNAs did not change the tested metabolome in a substantial way. The analysis revealed a fractionation of the data in the PCA into nonphosphorylated (negative PC2) and phosphorylated precursors (positive PC2). These sugars and sugar phosphates are direct precursors of the fucosylation pathway and might accumulate upon downregulation of FUK or FUT8. A shift in the score plot for cells expressing 2 x miR‐34a‐5p towards the FUT8 siRNA was observed, in contrast to the cells expressing only one copy of this miRNA. As the siRNA and miR‐34a‐5p both target and regulate FUT8, this might lead to similar subtle metabolic changes. On the contrary, the expression of the chained 2 x miR‐3096b‐5p plasmid led to a shift of the cells towards the mock cells, which would indicate more balanced and favourable levels of the selected metabolites. Generally, the observed metabolic shifts were subtle, and little is known about potential feedback loops concerning the whole glycosylation machinery. Summarized, our findings demonstrate the usability of miRNAs to induce stable gene regulation and fine‐tune phenotypic changes. For the purpose of biosimilar production, where new methods are needed to obtain the properties of the originator, chaining of miRNAs or the application of designed amiRNAs represents a promising new perspective. These methods enable a fine‐tuning of specific target genes leading to a more precise regulation of phenotypes, as we demonstrated here using the example of mAb fucosylation. However, further studies need to be conducted to conclusively decide whether increase of overexpression by miRNA chaining or development of more efficient amiRNAs based on native miRNAs should be the method of choice for a successful application of the miRNA technology.

## AUTHOR CONTRIBUTIONS

Kerstin Otte, Christian Neusüß, and Friedemann Hesse supervised the project. Patrick Schlossbauer, Florian Klingler, Lukas Naumann, and Madina Burkhart planned experiments and Patrick Schlossbauer conducted them. Patrick Schlossbauer, Lukas Naumann, and Kathrin Korff analyzed and evaluated data and Patrick Schlossbauer wrote the manuscript. René Handrick gave significant scientific advice. All authors read the final manuscript and provided critical feedback.

## CONFLICT OF INTEREST STATEMENT

The authors declare no conflicts of interest.

## Supporting information

Supporting Information
Supplement Inf. 1 Additional information regarding the metabolite analysis of CHO cells stably overexpressing selected miRNAs. Sample preparation, internal standards, and metabolite standards used for sample preparation and metabolite identification.

Supporting Information
Supplement Tab. 1 Sequences of miRNAs and artificial miRNAs (amiR) used in this study. Sequences are given from 5′ to 3′ end. Sequences were ordered as mimics for transient transfections or synthesized for cloning in the pcDNA6.2‐GW/EmGFP‐miR plasmid for stable transfection in a CHO cell line.

Supporting Information
Supplement Tab. 2 Primers used for the amplification of pre‐miR sequences of miR‐34a‐5p or miR‐3096b‐5p including ∼100 base pairs of up‐ and downstream flanking regions from murine genome for cloning into the pEGP‐miR plasmid system.

Supporting Inforamtion
Supplement Tab. 3 Degree of fucosylation, galactosylation and sialylation on the mAbs produced in the stable cell pools overexpressing miRNAs and artificial miRNAs (amiRNAs). X‐fold was calculated relative to the mock control.Supplement Information 1

Supporting Information

## Data Availability

The data that support the findings of this study are available from the corresponding author, PS, upon reasonable request.

## References

[1] Kim JY , Kim Y‐G , Lee GM . CHO cells in biotechnology for production of recombinant proteins: current state and further potential. Appl Microbiol Biotechnol. 2012;93:917‐930. doi:10.1007/s00253-011-3758-5 22159888

[2] Walsh G , Walsh E . Biopharmaceutical benchmarks 2022. Nat Biotechnol. 2022;40:1722‐1760. doi:10.1038/s41587-022-01582-x 36471135 PMC9735008

[3] Fischer S , Handrick R , Otte K . The art of CHO cell engineering: a comprehensive retrospect and future perspectives. Biotechnol Adv. 2015;33:1878‐1896. doi:10.1016/j.biotechadv.2015.10.015 26523782

[4] Edwards E , Livanos M , Krueger A , et al. Strategies to control therapeutic antibody glycosylation during bioprocessing: synthesis and separation. Biotechnol Bioeng. 2022;119:1343‐1358. doi:10.1002/bit.28066 35182428 PMC9310845

[5] Yamane‐Ohnuki N , Kinoshita S , Inoue‐Urakubo M , et al. Establishment of FUT8 knockout Chinese hamster ovary cells: an ideal host cell line for producing completely defucosylated antibodies with enhanced antibody‐dependent cellular cytotoxicity. Biotechnol Bioeng. 2004;87:614‐622. doi:10.1002/bit.20151 15352059

[6] Fischer S , Marquart KF , Pieper LA , et al. miRNA engineering of CHO cells facilitates production of difficult‐to‐express proteins and increases success in cell line development. Biotechnol Bioeng. 2017;114:1495‐1510. doi:10.1002/bit.26280 28262952 PMC6084326

[7] Wang P , Zhou Y , Richards AM . Effective tools for RNA‐derived therapeutics: siRNA interference or miRNA mimicry. Theranostics. 2021;11:8771‐8796. doi:10.7150/thno.62642 34522211 PMC8419061

[8] Loh WP , Loo B , Zhou L , et al. Overexpression of microRNAs enhances recombinant protein production in Chinese hamster ovary cells. Biotechnol J. 2014;9:1140‐1151. doi:10.1002/biot.201400050 24819042

[9] Bartel DP . MicroRNAs: target recognition and regulatory functions. Cell. 2009;136:215‐233. doi:10.1016/j.cell.2009.01.002 19167326 PMC3794896

[10] Brennecke J , Stark A , Russell RB , et al. Principles of microRNA‐target recognition. PLOS Biol. 2005;3:e85. doi:10.1371/journal.pbio.0030085 15723116 PMC1043860

[11] Filipowicz W , Bhattacharyya SN , Sonenberg N . Mechanisms of post‐transcriptional regulation by microRNAs: are the answers in sight? Nat Rev Genet. 2008;9:102‐114. doi:10.1038/nrg2290 18197166

[12] Pei Y , Tuschl T . On the art of identifying effective and specific siRNAs. Nat Methods. 2006;3:670‐676. doi:10.1038/nmeth911 16929310

[13] Krol J , Loedige I , Filipowicz W . The widespread regulation of microRNA biogenesis, function and decay. Nat Rev Genet. 2010;11:597‐610. doi:10.1038/nrg2843 20661255

[14] Gurtan AM , Sharp PA . The role of miRNAs in regulating gene expression networks. J Mol Biol. 2013;425:3582‐3600. doi:10.1016/j.jmb.2013.03.007 23500488 PMC3757117

[15] Hackl M , Borth N , Grillari J . miRNAs − pathway engineering of CHO cell factories that avoids translational burdening. Trends Biotechnol. 2012;30:405‐406. doi:10.1016/j.tibtech.2012.05.002 22673691

[16] Müller D , Katinger H , Grillari J . MicroRNAs as targets for engineering of CHO cell factories. Trends Biotechnol. 2008;26:359‐365. doi:10.1016/j.tibtech.2008.03.010 18471912

[17] Clarke C , Henry M , Doolan P , et al. Integrated miRNA, mRNA and protein expression analysis reveals the role of post‐transcriptional regulation in controlling CHO cell growth rate. BMC Genomics. 2012;13:656. doi:10.1186/1471-2164-13-656 23170974 PMC3544584

[18] Jadhav V , Hackl M , Bort JAH , et al. A screening method to assess biological effects of microRNA overexpression in Chinese hamster ovary cells. Biotechnol Bioeng. 2012;109:1376‐1385. doi:10.1002/bit.24490 22407745

[19] Strotbek M , Florin L , Koenitzer J , et al. Stable microRNA expression enhances therapeutic antibody productivity of Chinese hamster ovary cells. Metab Eng. 2013;20:157‐166. doi:10.1016/j.ymben.2013.10.005 24144501

[20] Fischer S , Buck T , Wagner A , et al. A functional high‐content miRNA screen identifies miR‐30 family to boost recombinant protein production in CHO cells. Biotechnol J. 2014;9:1279‐1292. doi:10.1002/biot.201400306 25061012

[21] Jadhav V , Hackl M , Klanert G , et al. Stable overexpression of miR‐17 enhances recombinant protein production of CHO cells. J Biotechnol. 2014;175:38‐44. doi:10.1016/j.jbiotec.2014.01.032 24518263 PMC3991393

[22] Fischer S , Mathias S , Schaz S , et al. Enhanced protein production by microRNA‐30 family in CHO cells is mediated by the modulation of the ubiquitin pathway. J Biotechnol. 2015;212:32‐43. doi:10.1016/j.jbiotec.2015.08.002 26256096

[23] Leroux A‐C , Bartels E , Winter L , et al. Transferability of miRNA‐technology to bioprocessing: influence of cultivation mode and media. Biotechnol Prog. 2021;37:e3107. doi:10.1002/btpr.3107 33300297 PMC8244005

[24] Stiefel F , Fischer S , Sczyrba A , et al. miRNA profiling of high, low and non‐producing CHO cells during biphasic fed‐batch cultivation reveals process relevant targets for host cell engineering. J Biotechnol. 2016;225:31‐43. doi:10.1016/j.jbiotec.2016.03.028 27002234

[25] Loh WP , Yang Y , Lam KP . miR‐92a enhances recombinant protein productivity in CHO cells by increasing intracellular cholesterol levels. Biotechnol J. 2017;12. doi:10.1002/biot.201600488 28146316

[26] Liu H‐N , Dong W‐H , Lin Y , et al. The effect of microRNA on the production of recombinant protein in CHO cells and its mechanism. Front Bioeng Biotechnol. 2022;10:832065. doi:10.3389/fbioe.2022.832065 35387297 PMC8977551

[27] Mori K , Kuni‐Kamochi R , Yamane‐Ohnuki N , et al. Engineering Chinese hamster ovary cells to maximize effector function of produced antibodies using FUT8 siRNA. Biotechnol Bioeng. 2004;88:901‐908. doi:10.1002/bit.20326 15515168

[28] Raymond C , Robotham A , Spearman M , et al. Production of α2,6‐sialylated IgG1 in CHO cells. mAbs. 2015;7:571‐583. doi:10.1080/19420862.2015.1029215 25875452 PMC4622614

[29] Weis BL , Guth N , Fischer S , et al. Stable miRNA overexpression in human CAP cells: engineering alternative production systems for advanced manufacturing of biologics using miR‐136 and miR‐3074. Biotechnol Bioeng. 2018;115:2027‐2038. doi:10.1002/bit.26715 29665036

[30] Singh A , Fan Y , Cakal S , et al. Identification of novel miRNA targets in CHO cell lines and characterization of their impact on protein N‐glycosylation. Authorea. Identification of novel miRNA targets in CHO cell lines and characterization of their impact on protein N‐glycosylation2022; doi: 10.22541/au,164167102.22702519/v1

[31] Malphettes L , Freyvert Y , Chang J , et al. Highly efficient deletion of FUT8 in CHO cell lines using zinc‐finger nucleases yields cells that produce completely nonfucosylated antibodies. Biotechnol Bioeng. 2010;106:774‐783. doi:10.1002/bit.22751 20564614

[32] Zhang P , Haryadi R , Chan KF , et al. Identification of functional elements of the GDP‐fucose transporter SLC35C1 using a novel Chinese hamster ovary mutant. Glycobiology. 2012;22:897‐911. doi:10.1093/glycob/cws064 22492235

[33] Yang Z , Wang S , Halim A , et al. Engineered CHO cells for production of diverse, homogeneous glycoproteins. Nat Biotechnol. 2015;33:842‐844. doi:10.1038/nbt.3280 26192319

[34] Louie S , Haley B , Marshall B , et al. FX knockout CHO hosts can express desired ratios of fucosylated or afucosylated antibodies with high titers and comparable product quality. Biotechnol Bioeng. 2017;114:632‐644. doi:10.1002/bit.26188 27666939

[35] Beuger V , Künkele K‐P , Koll H , et al. Short‐hairpin‐RNA‐mediated silencing of fucosyltransferase 8 in Chinese‐hamster ovary cells for the production of antibodies with enhanced antibody immune effector function. Biotechnol Appl Biochem. 2009;53:31‐37. doi:10.1042/BA20080220 19032154

[36] Imai‐Nishiya H , Mori K , Inoue M , et al. Double knockdown of alpha1,6‐fucosyltransferase (FUT8) and GDP‐mannose 4,6‐dehydratase (GMD) in antibody‐producing cells: a new strategy for generating fully non‐fucosylated therapeutic antibodies with enhanced ADCC. BMC Biotechnol. 2007;7:84. doi:10.1186/1472-6750-7-84 18047682 PMC2216013

[37] Klingler F , Naumann L , Schlossbauer P , et al. A novel system for glycosylation engineering by natural and artificial miRNAs. Metab Eng. 2023. doi:10.1016/j.ymben.2023.03.004 36906118

[38] Allen JG , Mujacic M , Frohn MJ , et al. Facile modulation of antibody fucosylation with small molecule fucostatin inhibitors and cocrystal structure with GDP‐mannose 4,6‐dehydratase. ACS Chem Biol. 2016;11:2734‐2743. doi:10.1021/acschembio.6b00460 27434622

[39] Pijnenborg JFA , Visser EA , Noga M , et al. Cellular fucosylation inhibitors based on fluorinated fucose‐1‐phosphates*. Chemistry. 2021;27:4022‐4027. doi:10.1002/chem.202005359 33336886 PMC7986151

[40] Mishra N , Spearman M , Donald L , et al. Comparison of two glycoengineering strategies to control the fucosylation of a monoclonal antibody. J Biotechnol. 2020;324S:100015. doi:10.1016/j.btecx.2020.100015 34154738

[41] Joubert S , Guimond J , Perret S , et al. Production of afucosylated antibodies in CHO cells by coexpression of an anti‐FUT8 intrabody. Biotechnol Bioeng. 2022;119:2206‐2220. doi:10.1002/bit.28127 35509261

[42] Ha M , Kim VN . Regulation of microRNA biogenesis. Nat Rev Mol Cell Biol. 2014;15:509‐524. doi:10.1038/nrm3838 25027649

[43] Murchison EP , Hannon GJ . miRNAs on the move: miRNA biogenesis and the RNAi machinery. Curr Opin Cell Biol. 2004;16:223‐229. doi:10.1016/j.ceb.2004.04.003 15145345

[44] Starega‐Roslan J , Koscianska E , Kozlowski P , et al. The role of the precursor structure in the biogenesis of microRNA. Cell Mol Life Sci. 2011;68:2859‐2871. doi:10.1007/s00018-011-0726-2 21607569 PMC3155042

[45] Lee Y , Kim M , Han J , et al. MicroRNA genes are transcribed by RNA polymerase II. EMBO J. 2004;23:4051‐4060. doi:10.1038/sj.emboj.7600385 15372072 PMC524334

[46] Kim VN , Han J , Siomi MC . Biogenesis of small RNAs in animals. Nat Rev Mol Cell Biol. 2009;10:126‐139. doi:10.1038/nrm2632 19165215

[47] O'Brien J , Hayder H , Zayed Y , et al. Overview of MicroRNA biogenesis, mechanisms of actions, and circulation. Front Endocrinol (Lausanne). 2018;9:402. doi:10.3389/fendo.2018.00402 30123182 PMC6085463

[48] Barron N , Kumar N , Sanchez N , et al. Engineering CHO cell growth and recombinant protein productivity by overexpression of miR‐7. J Biotechnol. 2011;151:204‐211. doi:10.1016/j.jbiotec.2010.12.005 21167223

[49] Meleady P , Gallagher M , Clarke C , et al. Impact of miR‐7 over‐expression on the proteome of Chinese hamster ovary cells. J Biotechnol. 2012;160:251‐262. doi:10.1016/j.jbiotec.2012.03.002 22445466

[50] Jin HY , Gonzalez‐Martin A , Miletic AV , et al. Transfection of microRNA Mimics Should Be Used with Caution. Front Genet. 2015;6:340. doi:10.3389/fgene.2015.00340 26697058 PMC4667072

[51] Søkilde R , Newie I , Persson H , et al. Passenger strand loading in overexpression experiments using microRNA mimics. RNA Biol. 2015;12:787‐791. doi:10.1080/15476286.2015.1020270 26121563 PMC4615182

[52] Klanert G , Jadhav V , Chanoumidou K , et al. Endogenous microRNA clusters outperform chimeric sequence clusters in Chinese hamster ovary cells. Biotechnol J. 2014;9:538‐544. doi:10.1002/biot.201300216 24323929 PMC4282078

[53] Lee JS , Park JH , Ha TK , et al. Revealing key determinants of clonal variation in transgene expression in recombinant CHO cells using targeted genome editing. ACS Synthetic Biology. 2018;7:2867‐2878. doi:10.1021/acssynbio.8b00290 30388888 PMC6535434

[54] Hilliard W , Lee KH . Systematic identification of safe harbor regions in the CHO genome through a comprehensive epigenome analysis. Biotechnol Bioeng. 2021;118:659‐675. doi:10.1002/bit.27599 33049068

[55] Kim M , O'Callaghan PM , Droms KA , et al. A mechanistic understanding of production instability in CHO cell lines expressing recombinant monoclonal antibodies. Biotechnol Bioeng. 2011;108:2434‐2446. doi:10.1002/bit.23189 21538334

[56] Liu C , Dalby B , Chen W , et al. Transient transfection factors for high‐level recombinant protein production in suspension cultured mammalian cells. Mol Biotechnol. 2008;39:141‐153. doi:10.1007/s12033-008-9051-x 18327552

[57] Bollin F , Dechavanne V , Chevalet L . Design of Experiment in CHO and HEK transient transfection condition optimization. Protein Expr Purif. 2011;78:61‐68. doi:10.1016/j.pep.2011.02.008 21354312

[58] Fischer S , Wagner A , Kos A , et al. Breaking limitations of complex culture media: functional non‐viral miRNA delivery into pharmaceutical production cell lines. J Biotechnol. 2013;168:589‐600. doi:10.1016/j.jbiotec.2013.08.027 23994267

[59] Myburgh R , Cherpin O , Schlaepfer E , et al. Optimization of critical hairpin features allows miRNA‐based gene knockdown upon single‐copy transduction. Mol Ther Nucleic Acids. 2014;3:e207. doi:10.1038/mtna.2014.58 25350582 PMC4217082

[60] Rousset F , Salmon P , Bredl S , et al. Optimizing synthetic miRNA minigene architecture for efficient miRNA hairpin concatenation and multi‐target gene knockdown. Mol Ther Nucleic Acids. 2019;14:351‐363. doi:10.1016/j.omtn.2018.12.004 30665184 PMC6350225

[61] Fowler DK , Williams C , Gerritsen AT , et al. Improved knockdown from artificial microRNAs in an enhanced miR‐155 backbone: a designer's guide to potent multi‐target RNAi. Nucleic Acids Res. 2016;44:e48. doi:10.1093/nar/gkv1246 26582923 PMC4797272

[62] Bourhill T , Arbuthnot P , Ely A . Successful disabling of the 5' UTR of HCV using adeno‐associated viral vectors to deliver modular multimeric primary microRNA mimics. J Virol Methods. 2016;235:26‐33. doi:10.1016/j.jviromet.2016.05.008 27181212

[63] Sun D , Melegari M , Sridhar S , et al. Multi‐miRNA hairpin method that improves gene knockdown efficiency and provides linked multi‐gene knockdown. Biotechniques. 2006;41:59‐63. doi:10.2144/000112203 16869514

[64] Idogawa M , Sasaki Y , Suzuki H , et al. A single recombinant adenovirus expressing p53 and p21‐targeting artificial microRNAs efficiently induces apoptosis in human cancer cells. Clin Cancer Res. 2009;15:3725‐3732. doi:10.1158/1078-0432.CCR-08-2396 19458054

[65] Kang SG , Roh YM , Lau A , et al. Establishment and characterization of Prnp knockdown neuroblastoma cells using dual microRNA‐mediated RNA interference. Prion. 2011;5:93‐102. doi:10.4161/pri.5.2.15621 21494092 PMC3166508

[66] Peipp M , van Lammerts Bueren JJ , Schneider‐Merck T , et al. Antibody fucosylation differentially impacts cytotoxicity mediated by NK and PMN effector cells. Blood. 2008;112:2390‐2399. doi:10.1182/blood-2008-03-144600 18566325

[67] Rehberger B , Wodarczyk C , Reichenbächer B , et al. Accelerating stable recombinant cell line development by targeted integration. BMC Proc. 2013;7:1‐3. doi:10.1186/1753-6561-7-S6-P111

[68] Livak KJ , Schmittgen TD . Analysis of relative gene expression data using real‐time quantitative PCR and the 2(‐Delta Delta C(T)) method. Methods. 2001;25:402‐408. doi:10.1006/meth.2001.1262 11846609

[69] Naumann L , Schlossbauer P , Klingler F , et al. High‐throughput glycosylation analysis of intact monoclonal antibodies by mass spectrometry coupled with capillary electrophoresis and liquid chromatography. J Sep Sci. 2022;45:2034‐2044. doi:10.1002/jssc.202100865 35044720

[70] Naumann L , Haun A , Höchsmann A , et al. Augmented region of interest for untargeted metabolomics mass spectrometry (AriumMS) of multi‐platform‐based CE‐MS and LC‐MS data. Anal Bioanal Chem. 2023. doi:10.1007/s00216-023-04715-6 PMC10287804 37225900

[71] van den Berg RA , Hoefsloot HCJ , Westerhuis JA , et al. Centering, scaling, and transformations: improving the biological information content of metabolomics data. BMC Genomics. 2006;7:142. doi:10.1186/1471-2164-7-142 16762068 PMC1534033

[72] Ko P , Misaghi S , Hu Z , et al. Probing the importance of clonality: single cell subcloning of clonally derived CHO cell lines yields widely diverse clones differing in growth, productivity, and product quality. Biotechnol Prog. 2018;34:624‐634. doi:10.1002/btpr.2594 29226566

[73] Pilbrough W , Munro TP , Gray P . Intraclonal protein expression heterogeneity in recombinant CHO cells. PLoS One. 2009;4:e8432. doi:10.1371/journal.pone.0008432 20037651 PMC2793030

[74] Feichtinger J , Hernández I , Fischer C , et al. Comprehensive genome and epigenome characterization of CHO cells in response to evolutionary pressures and over time. Biotechnol Bioeng. 2016;113:2241‐2253. doi:10.1002/bit.25990 27072894 PMC5006888

[75] Wurm F , Wurm M . Cloning of CHO cells, productivity and genetic stability—a discussion. Processes. 2017;5:20. doi:10.3390/pr5020020

[76] Baskerville S , Bartel DP . Microarray profiling of microRNAs reveals frequent coexpression with neighboring miRNAs and host genes. RNA. 2005;11:241‐247. doi:10.1261/rna.7240905 15701730 PMC1370713

[77] Wang Y , Luo J , Zhang H , et al. microRNAs in the Same clusters evolve to coordinately regulate functionally related genes. Mol Biol Evol. 2016;33:2232‐2247. doi:10.1093/molbev/msw089 27189568 PMC4989102

[78] Kabekkodu SP , Shukla V , Varghese VK , et al. Clustered miRNAs and their role in biological functions and diseases. Biol Rev Camb Philos Soc. 2018;93:1955‐1986. doi:10.1111/brv.12428 29797774

[79] Altuvia Y , Landgraf P , Lithwick G , et al. Clustering and conservation patterns of human microRNAs. Nucleic Acids Res. 2005;33:2697‐2706. doi:10.1093/nar/gki567 15891114 PMC1110742

[80] Cheng L , Gao S , Song X , et al. Comprehensive N‐glycan profiles of hepatocellular carcinoma reveal association of fucosylation with tumor progression and regulation of FUT8 by microRNAs. Oncotarget. 2016;7:61199‐61214. doi:10.18632/oncotarget.11284 27533464 PMC5308645

[81] Bernardi C , Soffientini U , Piacente F , et al. Effects of microRNAs on fucosyltransferase 8 (FUT8) expression in hepatocarcinoma cells. PLoS One. 2013;8:e76540. doi:10.1371/journal.pone.0076540 24130780 PMC3793929

