# Supplementary material for: Stable overexpression of native and artificial miRNAs for the production of differentially fucosylated antibodies in CHO cells
Source: Eng Life Sci. 2024 Apr 1;24(6):2300234. doi: 10.1002/elsc.202300234 (PMC11151017; doi:10.1002/elsc.202300234)
Supplement: Supplementary file 1 — Supporting Information Supplement Inf. 1 Additional information regarding the metabolite analysis of CHO cells stably overexpressing selected miRNAs. Sample preparation, internal standards, and metabolite standards used for sample preparation and metabolite identification. [file ELSC-24-2300234-s002.pdf]

Supplement Information 1

Stable overexpression of native and artificial miRNAs for the production of differentially fucosylated antibodies in CHO cells

1.1 Sample preparation

The lyophilized cells (8 mg) were dissolved in 485 µL ice-cold methanol/purified water (50/50, v/v) + 15 µL internal standard stock solution (1 mM), and disrupted with glass beads. After 15 min centrifugation, the non-soluble cellular components were removed by ultrafiltration. Lipids were removed using liquid-liquid extraction, by adding ice-cold 350 µL chloroform to the flow-through of the ultrafiltration. After vortexing and a 10 min incubation on ice, the sample was centrifuged and the non-polar chloroform phase was discarded. This extraction was repeated three times and the aqueous phase was dried afterwards within a Savant DNA 120 SpeedVac concentrator (Fisher Scientific GmbH, Schwerte, Germany). The dried sample was dissolved in 100 µL purified water for the HILIC-MS measurements. An Agilent 1100 Series LC-System (Agilent Technologies, Santa Clara, California, US) equipped with a VDSpher PUR 100 HILIC guard- and separation column (4.2 x 10 mm and 150 x 3 mm, 5 µm particle size, VDS optilab Chromatographiechnik GmbH, Berlin, Germany) thermostated at 30 °C was used. The LC was coupled to the Agilent 6510 QTOF with the respective standard dual ESI source and sprayer. The parameters for the QTOF were set to -3500 V spray voltage, 325 °C (8 L/min) dry gas temp (flow), 45 psig nebulizer, and 150/65/650 V for fragmentor/skimmer/octopole 1RF. The MS scan range was set to 100-17000 m/z (4

GHz high resolution), and for MS/MS experiments, ions were isolated with 4 amu (medium) and scanned from 50 to 1500 m/z.

1.2 Internal standard for sample preparation

15 µL ISTD (1 mM)

The 1 mM internal standard mixture consited out of the following components: benzene sulfonic acid, 2-nitrobenzoic acid, methionine sulfone, pentetic acid.

1.3 QC metabolite standard

Metabolite standard consists of pure substances diluted with purified water (origin of substances given in the materials section). The standard was pipetted together out of 4 stock solutions with a concentration of 1 mM: internal standards (4), sugars (nucleotide sugars, hexose-phosphates, hexoses) (12). The used standard concentrations of the mixture were 100 and 25 µM. The 100 µM concentration was used for all evaluations of this publication. The compounds were the following: internal standards: benzene sulfonic acid, 2-nitrobenzoic acid, methionine sulfone, pentetic acid; sugars: D-glucose, D-mannose, D-galactose, α-D-glucose-1-phosphate, D-fructose-1-phosphate, α-D-galactose-1-phosphate, α-D-mannose-1-phosphate.
