# Supplementary material for: Stable overexpression of native and artificial miRNAs for the production of differentially fucosylated antibodies in CHO cells
Source: Eng Life Sci. 2024 Apr 1;24(6):2300234. doi: 10.1002/elsc.202300234 (PMC11151017; doi:10.1002/elsc.202300234)
Supplement: Supplementary file 2 — Supporting Information Supplement Tab. 1 Sequences of miRNAs and artificial miRNAs (amiR) used in this study. Sequences are given from 5′ to 3′ end. Sequences were ordered as mimics for transient transfections or synthesized for cloning in the pcDNA6.2‐GW/EmGFP‐miR plasmid for stable transfection in a CHO cell line. [file ELSC-24-2300234-s001.pdf]

Supplement Table 1

**miRNA**	**Sequence 5‘ → 3‘**
miR-34a-5p	UGGCAGUGUCUUAGCUGGUUGU
miR-3096b-5p	GGCCAAGGAUGAGAACUCUAA
miR-3062-3p	CGGGGACACACUUUCUCCU
miR-669h-5p	AUGCAUGGGUGUAUAGUUGAGUGC
amiR-669h-1	UUGCAGAGGGCAGGAUGCAGUGUU
amiR-34a-1	UGGCAGUGUCUUUUCAUGGGGAC
amiR-34a-2	UGGCAGUGUCUUACAUGGGGAC
