# Supplementary material for: Stable overexpression of native and artificial miRNAs for the production of differentially fucosylated antibodies in CHO cells
Source: Eng Life Sci. 2024 Apr 1;24(6):2300234. doi: 10.1002/elsc.202300234 (PMC11151017; doi:10.1002/elsc.202300234)
Supplement: Supplementary file 3 — Supporting Information Supplement Tab. 2 Primers used for the amplification of pre‐miR sequences of miR‐34a‐5p or miR‐3096b‐5p including ∼100 base pairs of up‐ and downstream flanking regions from murine genome for cloning into the pEGP‐miR plasmid system. [file ELSC-24-2300234-s005.pdf]

Supplement Table 2

Primer Design pEGP

Mmu-miR-3096b	Sequence (5'->3')	Template strand	Length	Start	Stop	Tm	GC%	Self complementarity	Self 3' complementarity
1									
**Forward primer**	CTTGTAACAGTGCTGGTTCTTTTT	Plus	24	135	158	58.35	37.50	5.00	0.00
**Reverse primer**	ACCAGGTCCCAGAATCAAAGG	Minus	21	452	432	59.64	52.38	5.00	0.00
**Product length**	318								
2									
**Forward primer**	CTGCTTGTAACAGTGCTGGTTCTT	Plus	24	132	155	61.75	45.83	5.00	0.00
**Reverse primer**	TCTCTCCACCAGGTCCCAGA	Minus	20	459	440	61.44	60.00	5.00	3.00
**Product length**	328								

Mmu-miR-34a	Sequence (5'->3')	Template strand	Length	Start	Stop	Tm	GC%	Self complementarity	Self 3' complementarity
1									
**Forward primer**	CAGCTTCCAAAGTCCTGGCG	Plus	20	138	157	61.58	60.00	4.00	2.00
**Reverse primer**	CTCAGCTTTCTCCCATAGCGT	Minus	21	441	421	59.86	52.38	4.00	0.00
**Product length**	304								
2									
**Forward primer**	TTCCAAAGTCCTGGCGCCTC	Plus	20	142	161	62.75	60.00	6.00	2.00
**Reverse primer**	CAGCTTTCTCCCATAGCGTCC	Minus	21	439	419	60.81	57.14	4.00	0.00
**Product length**	298								
