# Supplementary material for: Stable overexpression of native and artificial miRNAs for the production of differentially fucosylated antibodies in CHO cells
Source: Eng Life Sci. 2024 Apr 1;24(6):2300234. doi: 10.1002/elsc.202300234 (PMC11151017; doi:10.1002/elsc.202300234)
Supplement: Supplementary file 4 — Supporting Inforamtion Supplement Tab. 3 Degree of fucosylation, galactosylation and sialylation on the mAbs produced in the stable cell pools overexpressing miRNAs and artificial miRNAs (amiRNAs). X‐fold was calculated relative to the mock control.Supplement Information 1 [file ELSC-24-2300234-s003.pdf]

Supplement Table 3

Stable cell pool	Fucosylation (x-fold to Mock)	Galactosylation (x-fold to Mock)	Sialylation (x-fold to Mock)
**Mock**	**1.0**	**1.0**	**1.0**
miR-34a-5p	0.48	1.13	1.1
2xmiR-34a-5p	0.47	1.11	1.2
4xmiR-34a-5p	0.45	1.04	0.96
miR-3096b-5p	0.64	1.08	1.08
2xmiR-3096b-5p	0.55	1.08	0.89
4xmiR-3096b-5p	0.38	1.04	0.92
miR-3062-3p	0.51	1.02	0.99
miR-669h-5p	0.69	1.02	1.05
amiR-669h-1	0.43	1.03	1.25
amiR-34a-1	0.19	0.89	0.9
amiR-34a-2	0.19	0.89	0.9
FUT8 siRNA	0.12	1.07	1.02
