# Supplementary material for: Stable overexpression of native and artificial miRNAs for the production of differentially fucosylated antibodies in CHO cells
Source: Eng Life Sci. 2024 Apr 1;24(6):2300234. doi: 10.1002/elsc.202300234 (PMC11151017; doi:10.1002/elsc.202300234)

Supplement Figure 1

**Supplement Fig. 1** LC-MS spectra of intact monoclonal antibody (mAb) produced from stable cell pools. The presented stable cell pools contain plasmids harbouring **A** 1x, 2x or 4xmiR-34a-5p **B** 1x, 2, or 4xmiR-3096b-5p **C** miR-669h-5p, amiR-34a-1, amiR-34a-2 or amiR-669h-1 **D** FUT8 siRNA. For comparison, all spectra contain a mock reference spectrum and all spectra are normalized to the G0F/G1F peak of the mock reference.


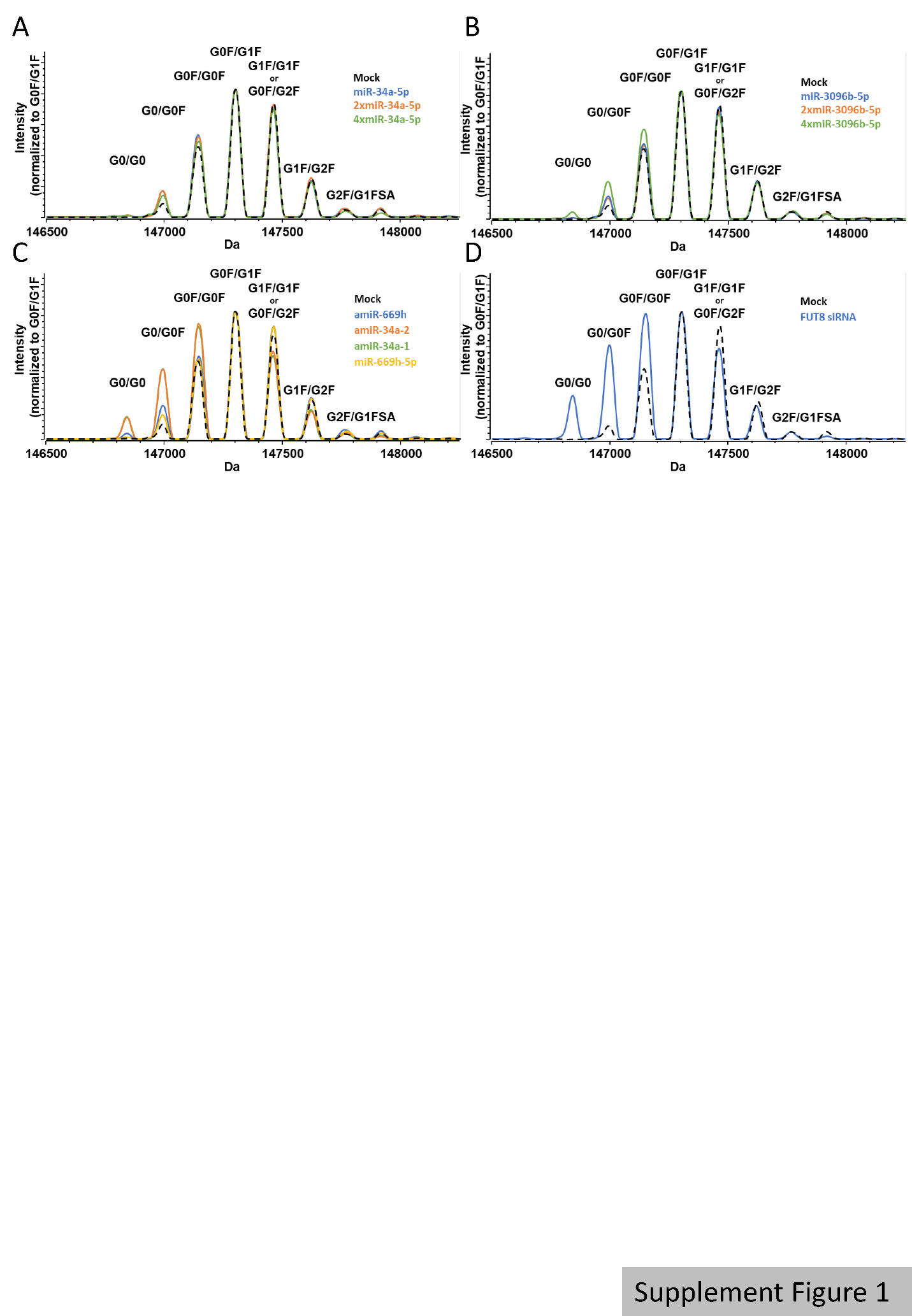


Supplement Figure 2

**Supplement Fig. 2** Analysis of antibiotic selection process for stable miRNA and respective artificial miRNAS (amiRNA) expression via the pcDNA6.2-GW/EmGFP-miR plasmid system. **A** Viability of selected example plasmids harbouring miRNAs, a H_2_O-control, and a mock plasmid was assayed via trypan blue exclusion after transfection. Blasticidin was added on day two and cells were passaged every two to three days with fresh medium containing the antibiotic. **B** Signal for green fluorescence protein (GFP) expression was measured at the passaging days via a flow cytometer. **C** Monoclonal antibody (mAb) fucosylation was measured on the same sample points. Data are presented as calculated normalized relative shares for every glycoform and relative to mock for every sample point.


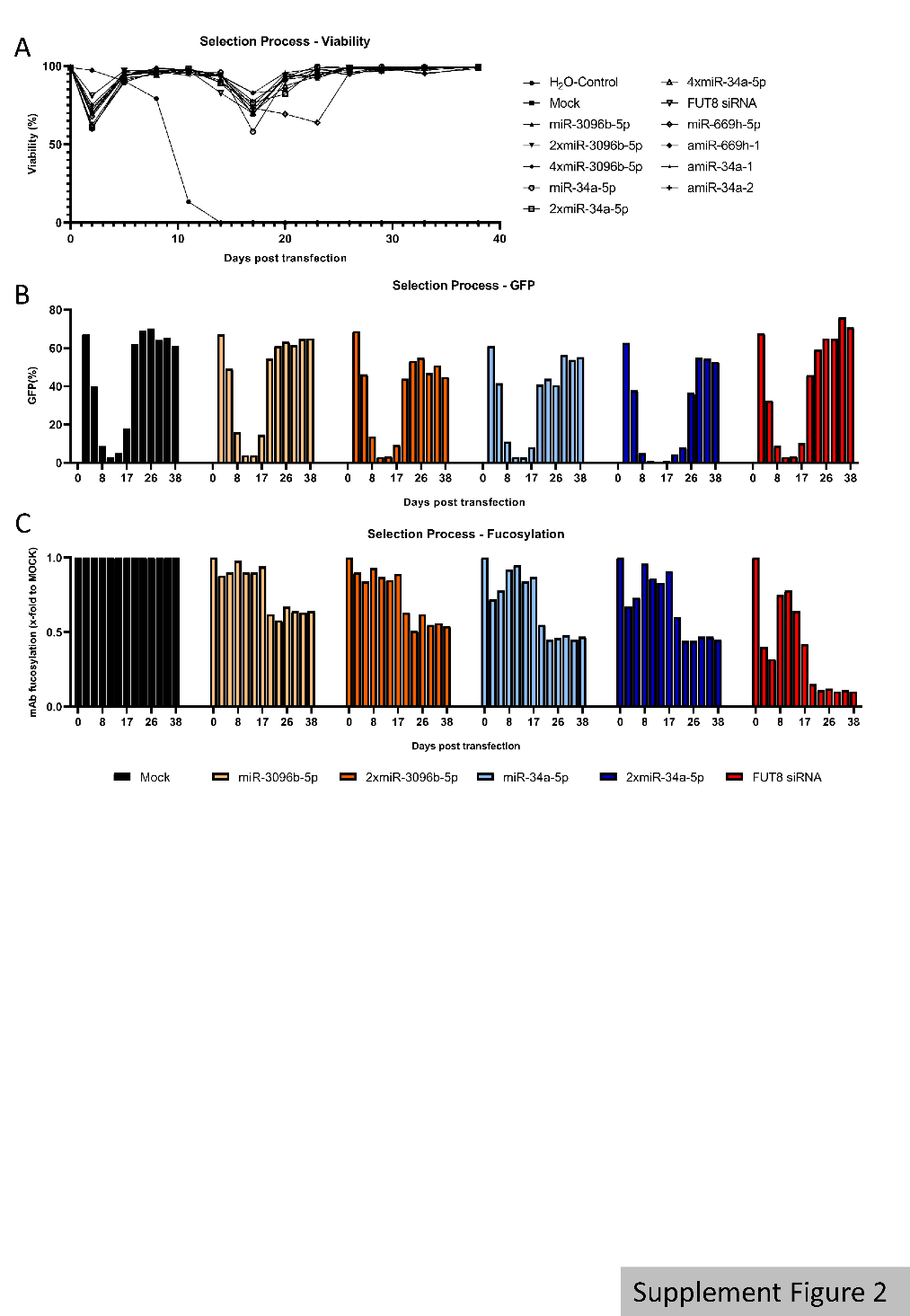


Supplement Figure 3

**Supplement Fig. 3** Single cell cloning of stable miR-3096b-5p pool cloned in the pcDNA6.2-GW/EmGFP-miR. **A** Comparison of overexpression of mature miR-3096b-5p in the cell pool and the single cell lines compared to MOCK (n = 3 biological replicates, mean + SD).


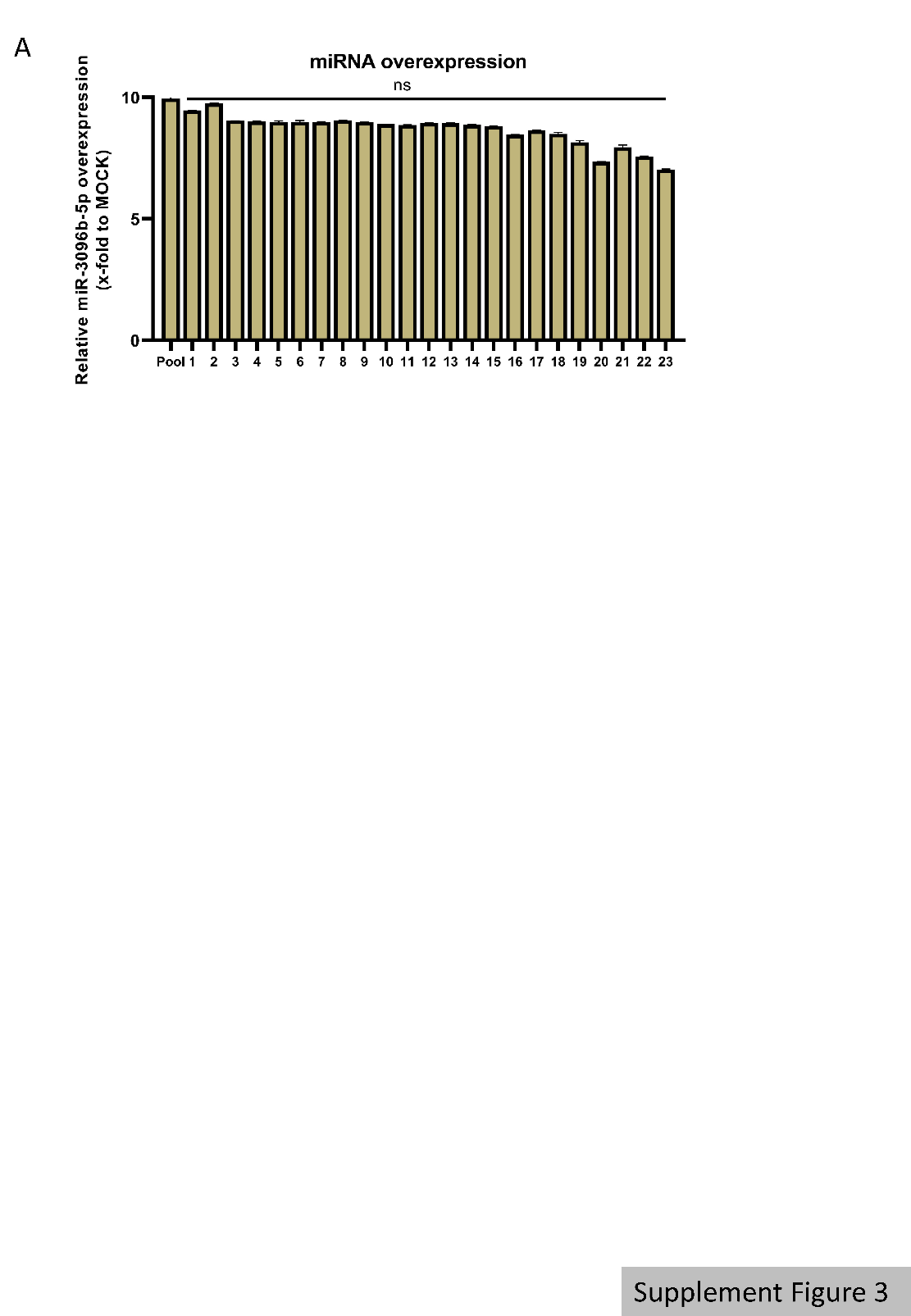

Supplement: Supplementary file 5 — Supporting Information [file ELSC-24-2300234-s004.docx]
